# IL‐4‐JAK1‐STAT6 Pathway Mediates Electroacupuncture's Effect on Microglial M2 Polarization to Treat Inflammatory Bowel Disease With Comorbid Depression

**DOI:** 10.1111/cns.70572

**Published:** 2025-08-18

**Authors:** Sihui Cao, Jingjing Yang, Lin Chen, Zuqiang Li, Lv Jia, Yuxiang Huang, Zhanwei Xu, Penghui Lu, Jin Liu, Qiong Liu, Mi Liu

**Affiliations:** ^1^ School of Acupuncture‐Moxibustion, Tuina and Rehabilitation Hunan University of Chinese Medicine Changsha Hunan China; ^2^ Department of Psychiatry, National Clinical Research Center for Mental Disorders, and National Center for Mental Disorders The Second Xiangya Hospital of Central South University Changsha Hunan China

**Keywords:** central nervous system inflammation, depression, electroacupuncture, inflammatory bowel disease, interleukin‐4, microglia

## Abstract

**Aims:**

Depression is prevalent in inflammatory bowel disease (IBD) and linked to neuroinflammation. However, the underlying mechanisms remain unclear. Therefore, we investigated the efficacy of electroacupuncture in mice with IBD and depression.

**Methods:**

An IBD mouse model of depression was established using 2.0% dextran sodium sulfate (DSS). After electroacupuncture, general condition and behavior were evaluated. Colon morphology was observed using hematoxylin and eosin staining. Serum inflammatory factors were detected using enzyme‐linked immunosorbent assay. Microglial activation was measured using immunofluorescence. Hippocampal protein expression was assessed using Western blotting and real‐time fluorescence quantitative polymerase chain reaction.

**Results:**

DSS‐induced model mice exhibited significant depression‐like behaviors and colon pathology. Serum and colon IL‐1β expression was elevated (*p* < 0.01), while IL‐4, IL‐10, and TGF‐β1 expression was decreased (*p* < 0.01 or *p* < 0.05). Hippocampal microglial activation was evident, with increased IL‐1β expression (*p* < 0.01) and reduced IL‐4, IL‐10, TGF‐β1, JAK1, STAT6, and p‐STAT6 expression (*p* < 0.01 or *p* < 0.05). Electroacupuncture resolved these changes; though its effects were significantly weakened after IL‐4 and p‐STAT6 inhibitor administration (*p* < 0.01 or *p* < 0.05). Transcriptomic sequencing of hippocampal tissue indicates that pro‐inflammatory pathways such as TNF/NF‐κB/mTOR are activated in the model group. Electroacupuncture can activate the IL‐4–mediated JAK–STAT signaling pathway, inhibit the activation of pro‐inflammatory signaling pathways, upregulate neuroprotective genes such as Slc2a3, Mef2d, and Jak1, and exert anti‐inflammatory effects.

**Conclusion:**

IL‐4‐JAK1‐STAT6 signaling may be an important pathway in mediating the efficacy of electroacupuncture in IBD with comorbid depression, particularly promoting microglial M2 polarization and improving neuroinflammation.

## Graphical abstract

A 2.0% dextran sodium sulfate (DSS) protocol was utilized to induce an inflammatory bowel disease (IBD) model accompanied by depression. In this model, mice demonstrated a significant elevation in the expression of pro‐inflammatory cytokines within the gastrointestinal tract, heightened levels of inflammatory markers in the bloodstream, and a pronounced M1 polarization of microglia within the hippocampal region. These microglia were found to secrete pro‐inflammatory cytokines, including Interleukin‐1 beta (IL‐1β), Tumor Necrosis Factor‐alpha (TNF‐α), and Interleukin‐18 (IL‐18), indicating a pronounced neuroinflammatory response. After undergoing electroacupuncture treatment, there was a marked elevation in the levels of IL‐4 both in the peripheral and central systems. When IL‐4 attaches to its receptor, it triggers the JAK1‐STAT6 signaling pathway, which in turn stimulates the expression of GATA3 and Arg‐1. This process steers microglial cells toward an anti‐inflammatory M2 phenotype. Consequently, this transition leads to the secretion of anti‐inflammatory agents, including IL‐4, IL‐10, and TGF‐β1, effectively alleviating neuroinflammation.

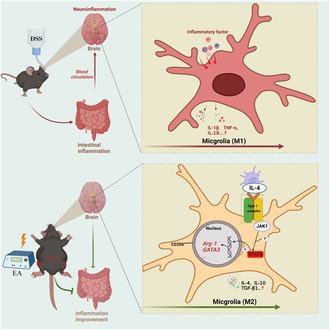

## 1 Introduction

Inflammatory bowel disease (IBD) encompasses a spectrum of disorders characterized by chronic and recurrent inflammation of the intestinal tract [1]. Although IBD primarily affects the intestines, its impact on emotional health should not be underestimated. Research has demonstrated that individuals with IBD are more likely to experience mental health issues, such as depression and anxiety, compared to those without the disease [2], with 25%–40% of patients reporting depressive symptoms [3, 4]. Moreover, depressive symptoms not only exacerbate IBD symptoms but also trigger relapses [5].

Recent research has confirmed the central role of inflammation in the pathological link between depression and IBD [6]. Patients with IBD exhibit a large proportion of abnormally secreted inflammatory mediators in the intestines, which can enter systemic circulation, penetrate the blood–brain barrier, activate microglia in the hippocampal region, trigger central nervous system inflammation, and ultimately lead to depressive symptoms [7, 8, 9, 10]. Abnormal microglial activation, which involves their transformation into the M1 phenotype, is a hallmark pathological feature of neuroinflammation [11]. Microglia, acting as regulators of brain homeostasis, adopt the classical M1 phenotype within inflammatory environments but revert to an alternatively activated M2 phenotype as inflammation resolves [12]. Consequently, promoting the shift of microglia towards the anti‐inflammatory M2 phenotype represents a promising strategy for alleviating IBD‐related depression.

Recent research has revealed that interleukin 4 (IL‐4) can prompt microglia to transition into the M2 phenotype, which is distinguished by elevated expression of arginase‐1 (Arg‐1) and CD206, as well as the secretion of anti‐inflammatory agents such as IL‐4, interleukin 10 (IL‐10), and transforming growth factor‐β1 (TGF‐β1) [13, 14]. This transition confers neuroprotective benefits, fostering neurogenesis and repairing damage within the hippocampal area, while also significantly mitigating inflammatory responses and reducing depressive symptoms [15]. Acupuncture has proved effective in alleviating excessive microglial activation [16, 17] and neuroinflammation [18, 19], thereby improving depressive symptoms. Elevated IL‐4 levels in rat serum and within the ischemic or reperfusion zones of the rat brain have also been reported following acupuncture [20, 21].

An expanding body of clinical and basic research validates the efficacy of acupuncture as an intervention for IBD with comorbid depression [16, 22]. Our previous research demonstrated that electroacupuncture at acupoints such as ST36 (Zusanli), ST25 (Tianshu), and LR3 (Taichong) can significantly reduce the Disease Activity Index (DAI) score in mice with IBD and comorbid depression while alleviating intestinal inflammatory responses. However, the underlying physiological mechanisms through which electroacupuncture ameliorates depressive symptoms in IBD remain unclear. Therefore, this study aimed to elucidate how electroacupuncture modulates the IL‐4‐JAK1‐STAT6 signaling pathway to promote the M2 phenotype in microglia, thereby effectively reducing neuroinflammation and improving depression‐like behaviors in mice with IBD and comorbid depression.

## 2 Methods

### 2.1 Animals

Seven‐week‐old male specific pathogen‐free (SPF) C57BL/6 mice were provided by Beijing Vital River Laboratory Animal Technology Co. Ltd. (license number: SCXK [Jing] 2021‐0006) and housed in the Animal Experiment Center of Hunan University of Chinese Medicine, with five mice per cage. The housing environment was maintained at a temperature of 20°C–25°C, humidity of 50%–70%, and a 12‐h light–dark cycle. All animal experimental protocols were approved by the Laboratory Animal Ethics Committee of Hunan University of Chinese Medicine (LL‐202209110002), and all procedures complied with the National Institutes of Health Guide for the Care and Use of Laboratory Animals.

### 2.2 Experimental Protocol and Groups

After a 7‐day acclimatization period, 68 male C57BL/6 mice were randomly divided into one of two groups: the control (CON) group (*n* = 14) and the modeling group (*n* = 54). The CON group was housed under standard conditions, whereas the modeling group was administered a 2.0% dextran sulfate sodium (DSS) solution (MP Biomedicals LLC) to induce IBD in a comorbid depression model [23, 24]. Upon successful model replication, the modeling group was further subdivided into five groups: the model group (MOD), electroacupuncture group (EA), IL‐4 inhibitor group (IL‐4 inhibitor), the electroacupuncture + IL‐4 inhibitor group (EA + IL‐4 inhibitor), and the combined medication group (Combination).

Thirty male C57BL/6 mice were used to establish an IBD model with comorbid depression. Upon successful model replication, the mice were randomly divided into EA, phosphorylated STAT6 (p‐STAT6) inhibitor, and the electroacupuncture + p‐STAT6 inhibitor groups (EA + *p*‐STAT6 inhibitor).

To further confirm the role of the IL‐4‐JAK1‐STAT6 signaling pathway in mediating the effects of electroacupuncture treatment on IBD with comorbid depression, 15 additional male C57BL/6 mice were adaptively fed for 7 days and then randomly divided into a control (CON) group (*n* = 5) and a modeling group (*n* = 10). Following successful modeling, the mice in the modeling group were further randomly divided into a model group (MOD) and an electroacupuncture group (EA) for intervention.

#### 2.2.1 Acupuncture Point Location

ST36, ST25, and LR3 were selected as acupuncture points for the electroacupuncture intervention. Acupuncture point locations were determined using the standard acupuncture point atlas for small animals outlined in the “14th Five‐Year Plan” national planning textbook Experimental Acupuncture Science and the anthropomorphic correspondence method [25, 26].

#### 2.2.2 Intervention Method

The EA, EA + IL‐4 inhibitor, and the EA + *p*‐STAT6 inhibitor groups were treated with model Φ0.18 × 13 mm acupuncture needles (Huatuo, Suzhou Medical Supplies Factory) inserted to a depth of approximately 2–3 mm at ST36, ST25, and LR3 acupoints. Electroacupuncture was conducted using a Han electroacupuncture device (Hans‐200A, Nanjing Jisheng Medical Technology Co. Ltd., Nanjing, China) [27], with a current of 1 mA, a frequency of 2/15 Hz, and dense‐sparse waves, lasting 20 min once daily for seven consecutive days.

**FIGURE 1:**
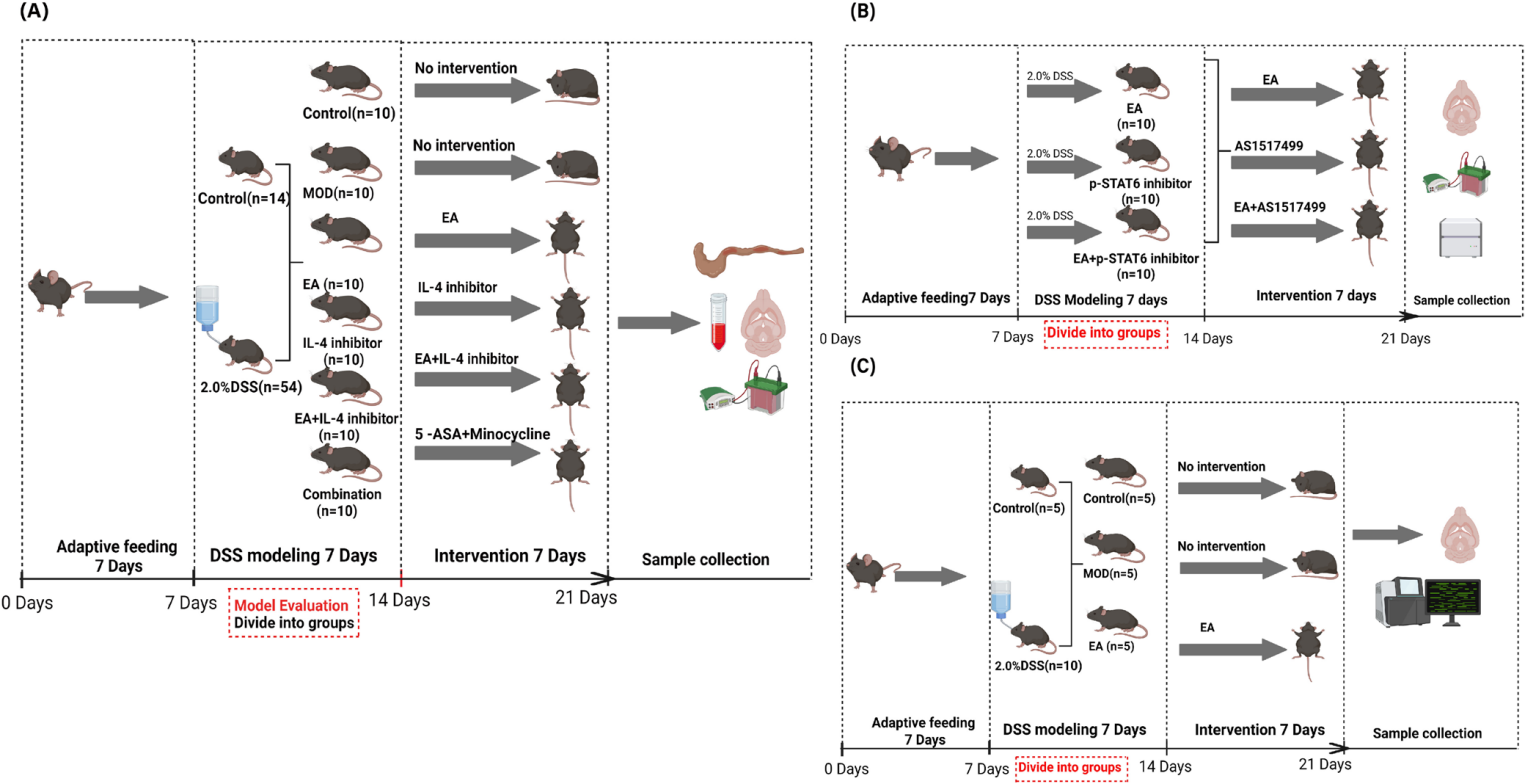
(A) Experimental design, part I. (B) Experimental design, part II. (C) Experimental design, part III.

The IL‐4 inhibitor and EA + IL‐4 inhibitor groups were intraperitoneally injected with an IL‐4 inhibitor (10 mg/kg; HY‐139092, MedChemExpress). The Combination group was intraperitoneally injected with 5‐aminosalicylic acid (200 mg/kg; Sigma, A3537) and minocycline (20 mg/kg; Sigma, M9511). The p‐STAT6 inhibitor and EA + *p*‐STAT6 inhibitor groups were intraperitoneally injected with a p‐STAT6 inhibitor (10 mg/kg; AS1517499; HY‐100614, MedChemExpress). The CON and MOD groups underwent the same handling procedure, isoflurane inhalation anesthesia, and intraperitoneal injection of an equivalent volume of physiological saline as the intervention groups. These interventions were conducted for seven continuous days (Figure 1).

### 2.3 Sample Collection and Processing

All samples were collected immediately after behavioral testing. Following intraperitoneal injection of 50 mg/kg of 1% pentobarbital sodium for anesthesia, the eyeballs were removed and approximately 1.5 mL of peripheral blood was collected in a centrifuge tube. The mice were then euthanized by cervical dislocation. Colon and hippocampal tissues were collected, with one portion fixed in 4% paraformaldehyde and another rapidly frozen in liquid nitrogen and stored at −80°C.

### 2.4 General Observation

Body weight, fecal consistency, occurrence of bloody stools, and DAI scores were monitored for each group following model induction [28, 29].

### 2.5 Behavioral Testing

#### 2.5.1 Forced Swim Test (FST)

Mice were individually placed into a transparent acrylic cylinder filled with water (25 cm height × 12 cm diameter), with the water temperature maintained at 23°C–25°C. Water depth was adjusted to ensure that the mouse's tails did not touch the bottom of the cylinder. After a 1‐min adaptation period, the duration of immobility within 4 min was recorded as an indicator of depression‐like behavior, with longer immobility periods indicating severe despair. Data were recorded and analyzed using AnyMaze software (Stoelting Company, USA) [30].

#### 2.5.2 Open Field Test (OFT)

A cubic gray open‐field box (55 cm side length) was divided into 16 equal squares, with the middle four blocks considered the central area. Mice were placed in the central area to start a 5‐min test, which included a 1‐min adaptation period that was not included in the analysis. The total distance traveled by the mice within 5 min and the time spent in the central area was recorded using AnyMaze software, with reduced movement and less time spent in the central area indicating higher anxiety and depression levels [31].

#### 2.5.3 Sucrose Preference Test (SPT)

Mice were housed individually for a 48‐h sucrose adaptation training period. Following water deprivation for 12 h, each mouse was provided with pure water and a 1% sucrose solution (200 mL each). After a further 12 h, the Sucrose Preference Rate (%) was calculated using the following formula: [Sucrose Solution Consumption / (Sucrose Solution Consumption + Water Consumption)] × 100%, with a sucrose preference rate < 0.4 or a significant difference from the CON group indicating anhedonia, an important symptom of depression [32].

### 2.6 Hematoxylin–Eosin Staining of Colon Tissue

Colon specimens fixed in 4% paraformaldehyde were thoroughly rinsed under running water, dehydrated using a graded series of alcohols, and cleared in xylene. The colon tissues were then embedded using a paraffin embedding machine. Once the paraffin solidified, the embedded colon tissues were sectioned into approximately 5 μm thick slices. After hematoxylin and eosin (HE) staining, the colon tissue morphology for each group was observed under different magnifications and fields of view using a light microscope. Pathological images were saved and assessed for pathological conditions.

### 2.7 Enzyme‐Linked Immunosorbent Assay for the Detection of Serum Cytokines IL‐1β, IL‐4, IL‐10, and TGF‐β1 Levels

Collected serum samples were left at room temperature for 1 h before centrifugation at 4°C for 15 min at 3000 *g* to separate the supernatant, which was then stored at −80°C. Enzyme‐linked immunosorbent assay (ELISA) detection kits and samples were then thawed at room temperature for 30 min. Mouse serum IL‐1β, IL‐4, IL‐10 (ED‐20174, ED‐20186, ED‐20162 from Xiamen Lunchangshuo Biotechnology Co. Ltd.), and TGF‐β1 (ab119557, Abcam) levels were detected using the respective ELISA kits.

### 2.8 Protein Expression Analysis by Western Blot in Colon and Hippocampal Tissues: IL‐1β, IL‐4, IL‐10, TGF‐β1, JAK1, STAT6, p‐STAT6

Colon and hippocampal tissues were homogenized, and total proteins were extracted using 200 μL of radioimmunoprecipitation assay lysis buffer. Protein concentrations were determined using a bicinchoninic acid protein assay kit. A 10% SDS‐PAGE gel (Bio‐Rad) was prepared for sample loading, electrophoresis, and subsequent transfer at 4°C. The membrane was blocked with 5% non‐fat dry milk for 1 h, washed, and then incubated with the following primary antibodies overnight at 4°C: IL‐4 (1:1000, AF5142, Affinity), IL‐10 (1:1000, DF6894, Affinity), TGF‐β1 (1:1000, AF1027, Affinity), IL‐1β (1:1000, AF5103, Affinity), JAK1 (1:2000, AF5012, Affinity), STAT6 (1:2000, AF6302, Affinity), and p‐STAT6 (1:2000, AF3301, Affinity). β‐actin antibody (1:1000, AF7018, Affinity) was used as a loading control. The membrane was washed three times with Tris‐buffered saline with Tween‐20 (TBST) for 10 min each. The membrane was then incubated with the secondary goat anti‐rabbit IgG H&L antibody (1:3000, S0001, Affinity) for 60 min. After three washes with TBST, the membrane was developed using an electrochemiluminescence (ECL) kit and a chemiluminescence imaging system (ChemiScope6100, Clinx Science Instruments Co. Ltd.). The grayscale values of the target bands were analyzed using ImageJ image processing software to determine the relative expression levels of the proteins of interest.

### 2.9 Real‐Time Fluorescence Quantitative Polymerase Chain Reaction Detection of IL‐4, Arg‐1, CD206, and GATA3 mRNA Expression in Hippocampal Tissue

Hippocampal tissues were collected, and total RNA was extracted using the TRIzol method according to the RNA extraction's instructions. The RNA was reverse‐transcribed into cDNA, which was then amplified. The following polymerase chain reaction (PCR) conditions were repeated for a total of 40 cycles: 50°C for 2 min; 95°C for 5 min; 95°C for 15 s, 60°C for 45 s. GAPDH was used as the reference gene, and the relative expression of the target genes was calculated using the $2^{-\Delta \Delta C_{t}}$ method. Primer sequences are listed in Table 1.

**TABLE 1:** Primer sequences.

Gene ID	Primer ID	Seq 5′‐3′	Amp size (bp)
IL‐4	F	GTCTCAACCCCCAGCTAGTT	120
	R	CTGTGACCTCGTTCAAAATGC	
GATA3	F	TTCCTCCGACCCCTTCTACT	216
	R	TTTTCACAGCACTAGAGACCCT	
CD206	F	CCTATGAAAATTGGGCTTACGG	131
	R	CTGACAAATCCAGTTGTTGAGG	
Arg‐1	F	GCAACCTGTGTCCTTTCTCCT	219
	R	GTCTCTTCCATCACCTTGCCA	

### 2.10 Hippocampal Tissue Immunofluorescence Staining

Hippocampal tissue sections were removed, warmed for 5 min, and rinsed three times with TBST at room temperature. After antigen retrieval at high temperature and pressure, the sections were blocked with 10% goat serum for 30 min. The sections were then incubated overnight at 4°C with the primary antibodies Iba‐1 (1:500, Abcam, ab178846) and CD206 (1:300, PTG, 18704‐1‐AP). After three washes with TBST, the sections were incubated with the fluorescent secondary goat anti‐rabbit IgG H&L antibody (1:500, Abcam, 150077) for 1 h in the dark. After three washes with TBST, the nuclei were counterstained with 4′,6‐diamidino‐2‐phenylindole (DAPI) (Solarbio, D8200) for 5 min. The sections were rinsed three times with TBST and mounted with an anti‐fluorescence quenching sealant. Images were captured under a fluorescence microscope, and positive expression areas were selected to calculate the average fluorescence intensity.

### 2.11 RNA Extraction, Library Construction, and Sequencing of Hippocampal Tissue

The TRIzol Reagent (Invitrogen, 15596‐018) was used to analyze frozen mice tissues, followed by total RNA isolation. The integrity and quality of RNA were assessed using the Agilent 2100 Bioanalyzer (Agilent Technologies, CA, USA) and RNase‐free agarose gel electrophoresis. The extracted mRNA was enriched using mRNA Capture Beads. After purification with beads, the mRNA was fragmented using high temperatures. The fragmented mRNA was then used as a template to synthesize the first strand of cDNA using a reverse transcription enzyme mixture. Although synthesizing the second strand of cDNA, end repair and A‐tailing were completed. Next, adapters were ligated, and Hieff NGS DNA Selection Beads were used for purification to select target fragments. PCR library amplification was then performed, followed by detection using the Illumina Nova Seq 6000 from Gene Denovo Biotechnology Co. (Guangzhou, China).

### 2.12 Statistical Analysis

Statistical analyses were performed using SPSS (version 25.0; IBM Corp., Armonk, NY) software. Quantitative data are expressed as the mean ± standard deviation. The Shapiro–Wilk test was used to assess the normality of the measurement data. One‐way ANOVA was used for intergroup comparisons for data that met the criteria for normality and homogeneity of variance, followed by post hoc Tukey pairwise comparisons, while non‐parametric Kruskal–Wallis tests were used for data that did not conform to a normal distribution or exhibited unequal variances. *p <* 0.05 were considered statistically significant. Graphs were generated using GraphPad Prism (version 8.0; GraphPad Software, San Diego, CA).

The raw data obtained through sequencing were stored in FASTQ format, and rigorous quality control measures were performed to ensure reliability and accuracy. The screening criteria for differentially expressed genes (DEGs) were set at a fold change > 1.5 and a significance *p* < 0.05. In GO analysis, terms with a *p* < 0.05 after false discovery rate (FDR) correction were considered significantly enriched. In KEGG analysis, terms with an adjusted *p* ≤ 0.05 were regarded as enriched. In GSEA analysis, a FDR < 0.25 was considered statistically significant. The bioinformatics analysis was completed using the real‐time interactive online data analysis platform Omicsmart (http://www.omicsmart.com).

## 3 Results

### 3.1 IBD Mice Exhibit Pronounced Depressive‐Like Behaviors

**FIGURE 2:**
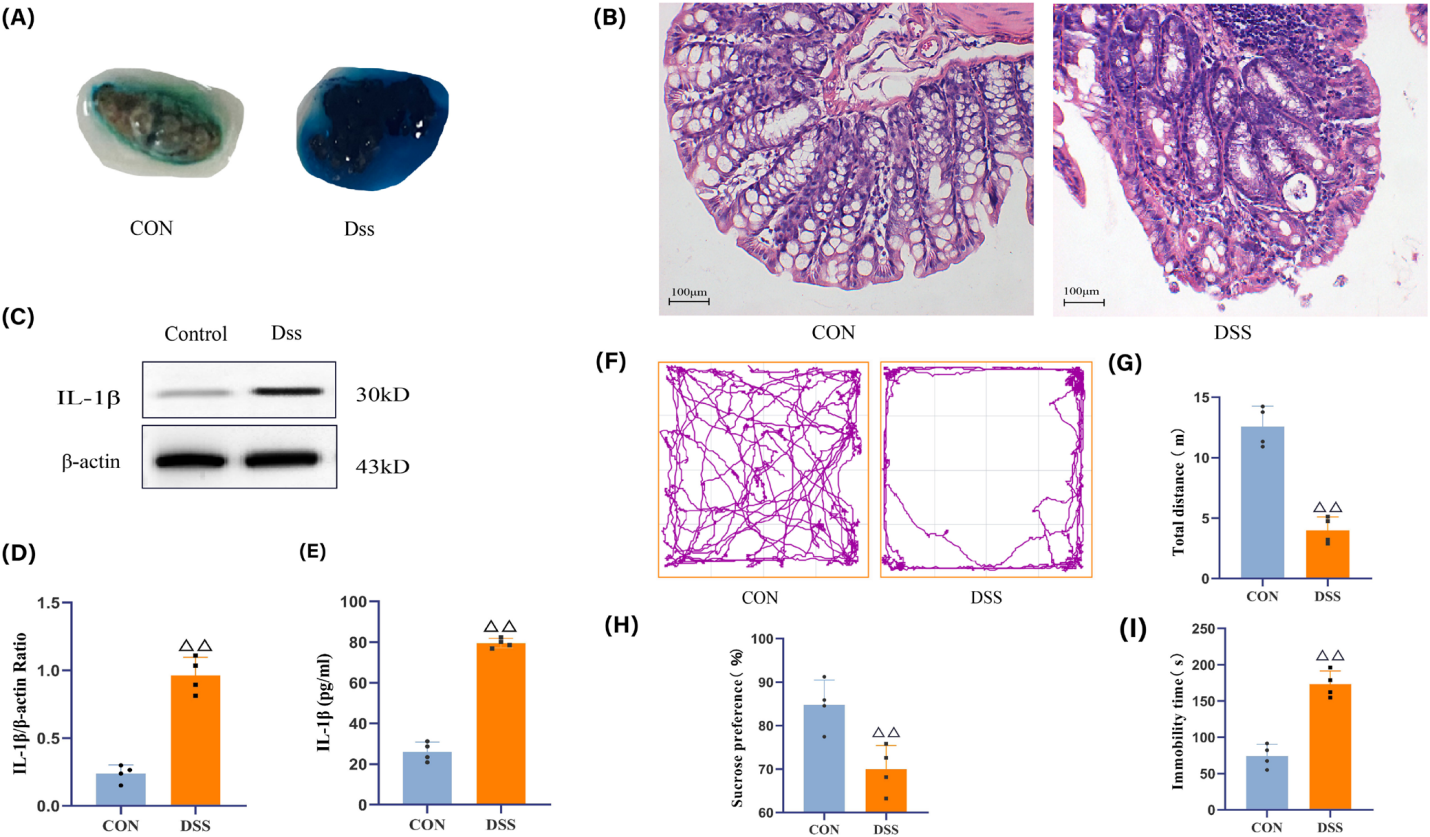
Model evaluation. (A) Results of fecal occult blood test in the CON group and DSS group post‐model induction. (B) HE staining of colon tissue from mice in the CON group and DSS group post‐model induction. The microscope has a magnification power of 200×, scale bar 100 μm. (C, D) Expression levels of IL‐1β in the colon tissue of mice from the CON group and DSS group post‐model induction. (E) Serum IL‐1β expression levels in mice from the CON group and DSS group post‐model induction. (F–I) Behavioral testing results for mice in the CON group and DSS group post‐model induction: (F) open‐field trajectory map of mice. (G) Total distance moved in the open field. (H) Sucrose preference percentage. (I) Immobility time in forced swimming test. *N* = 4 mice for each group. Compared with the CON group, ^△△^ *p <* 0.01.

Fecal occult blood tests revealed that the MOD group had positive or strongly positive fecal occult blood (Figure 2A). HE staining further confirmed that the colonic glandular structure of the CON group was intact, and the intestinal villi were smooth, whereas the MOD group exhibited intestinal wall edema, mucosal damage, and inflammatory cell infiltration (Figure 2B). Western Blot (WB) and ELISA results showed that, compared to the CON group, IL‐1β levels in the serum and colonic tissues of the MOD group mice were significantly increased (*p <* 0.05; Figure 2C–E). Subsequent behavioral tests (FST, OFT, and SPT) revealed that, compared to the CON group, the MOD group exhibited pronounced depressive‐like behaviors (Figure 2F–I).

### 3.2 Electroacupuncture Effectively Alleviates Colonic Inflammation in IBD Comorbid Depression Model Mice

**FIGURE 3:**
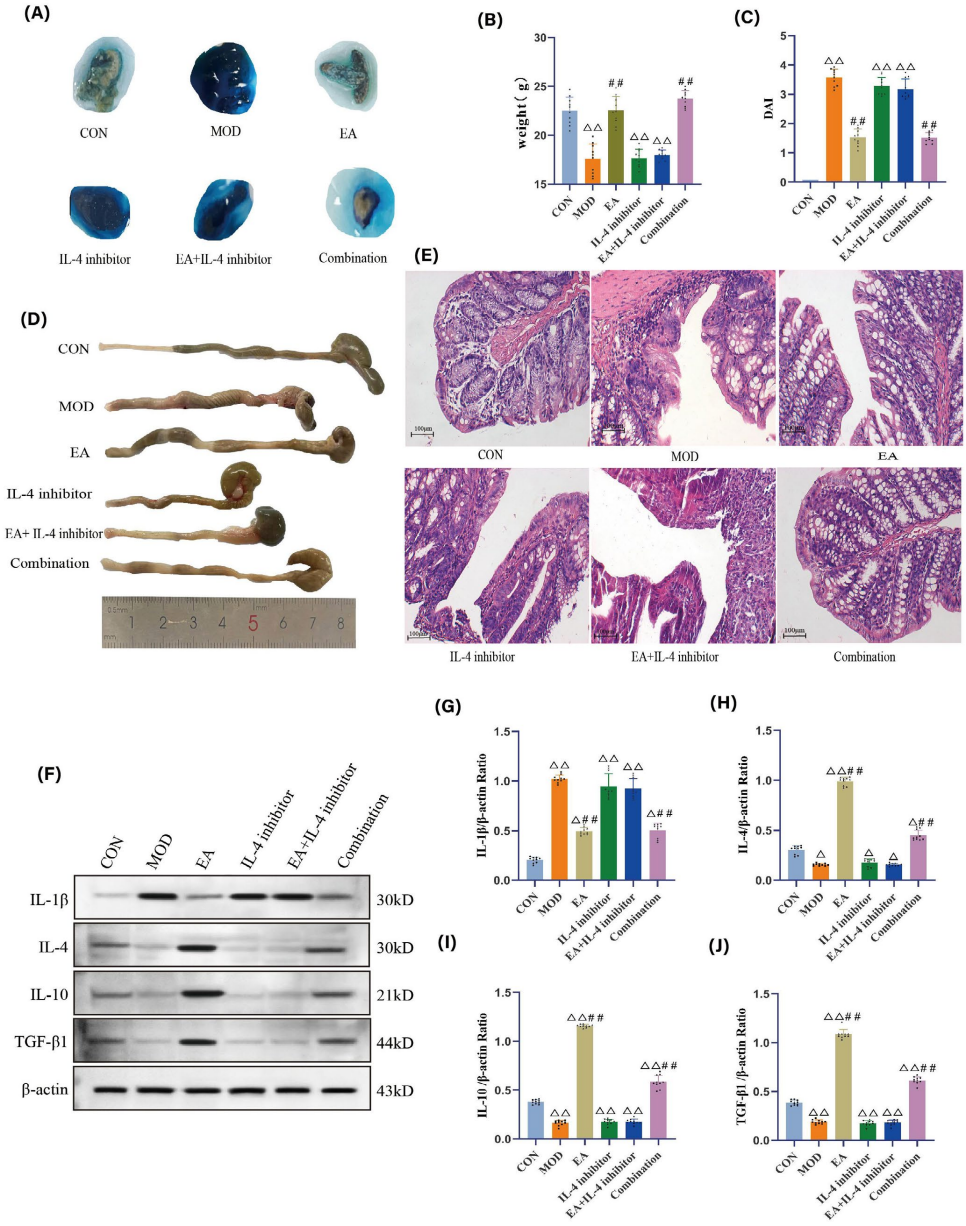
Detection results of IBD‐related indicators in mice of each group. (A) Post‐treatment results of fecal occult blood tests for each group. (B) Post‐treatment comparison of body weights among the different groups of mice. (C) Post‐treatment comparison of DAI scores across the various groups of mice. (D) Appearance and morphology of colon in each group of mice. (E) HE staining of colon tissue in each group of mice. The microscope has a magnification power of 200X, scale bar 100 μm. (G–J) Protein expression levels of IL‐1β, IL‐4, IL‐10, and TGF‐β1 in colon tissue of each group of mice. *N* = 10 mice for each group. Compared with the CON group, ^△^ *p <* 0.05, ^△△^ *p <* 0.01; compared with the MOD group, ^##^ *p <* 0.01.

The post‐intervention fecal occult blood test results are shown in Figure 3A. Compared to the CON group, most mice in the MOD, IL‐4 inhibitor, and EA + IL‐4 inhibitor groups tested positive (++), with some exhibiting strong positivity (+++). Compared to the MOD group, the occurrence of bloody stools in the EA and Combination groups significantly resolved, with most exhibiting weak positivity (+) and few exhibiting negativity (−).

Compared to the CON group, there was a significant reduction in body weight in the MOD, IL‐4 inhibitor, and EA + IL‐4 inhibitor groups (*p <* 0.01). Compared to the MOD group, there was a statistically significant increase in body weight in the EA and Combination groups (*p <* 0.01) (Figure 3B).

Compared to the CON group, there was a significant increase in DAI scores for the MOD, IL‐4 inhibitor, and EA + IL‐4 inhibitor groups (*p <* 0.01); whereas compared with the MOD group, the DAI scores in the EA group and the Combination group were significantly decreased (*p <* 0.01) (Figure 3C).

In the CON group, colons appeared normal in shape with smooth and shiny surfaces. The intestinal walls were intact with no edema, ulcers, perforations, or bleeding. In contrast, colon length in the MOD, IL‐4 inhibitor, and EA + IL‐4 inhibitor groups was significantly shortened, with turbid intestinal walls, congestion, edema, and loose fecal consistency within the intestine. Notably, the EA and Combination groups showed significant improvement in these symptoms (Figure 3D).

Colonic tissues in the MOD, IL‐4 inhibitor, and EA + IL‐4 inhibitor groups exhibited poor mucosal continuity, with destroyed glandular and epithelial tissue structures, disordered arrangement, inflammatory cell infiltration, edema, and bleeding. Additionally, the IL‐4 inhibitor group also exhibited a large number of shed goblet cells and necrotic, dissolved tissues. The EA and Combination groups showed better recovery of glandular structure and intestinal mucosal continuity, with a significant improvement in inflammatory conditions (Figure 3E).

Compared to the CON group, there was a significant increase in IL‐1β protein expression in colonic tissues in the MOD, IL‐4 inhibitor, and EA + IL‐4 inhibitor groups (*p <* 0.01). Although IL‐1β expression in the EA and Combination groups also increased (*p <* 0.05), it remained significantly lower than that in the MOD group (*p <* 0.01).

Compared to the CON group, the expression levels of IL‐4, IL‐10, and TGF‐β1 in the MOD, IL‐4 inhibitor, and EA + IL‐4 inhibitor groups were significantly reduced (*p <* 0.01 or *p <* 0.05). However, their expression in the EA and Combination groups was significantly higher than those in the CON (*p <* 0.01 or *p <* 0.05) and MOD groups (*p <* 0.01) (Figure 3F–J).

### 3.3 Electroacupuncture Reverses Depressive‐Like Behaviors in IBD Comorbid Depression Model Mice

**FIGURE 4:**
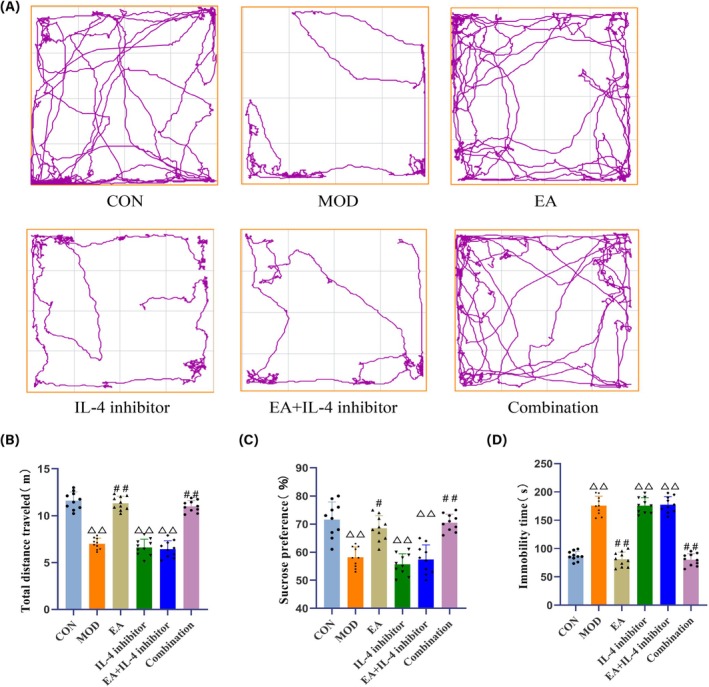
Behavioral test results of mice in each group. (A) Trajectory maps of open‐field tests for mice in each group. (B) Comparison of total distance moved in the open field after treatment for mice in each group. (C) Comparison of the percentage of sucrose preference after treatment for mice in each group. (D) Comparison of immobility time in forced swimming tests after treatment for mice in each group. *N* = 10 mice for each group. Compared with the CON group, ^△△^ *p <* 0.01; compared with the MOD group, ^#^ *p <* 0.05, ^##^ *p <* 0.01.

Compared to the CON group, the MOD, IL‐4 inhibitor, and EA + IL‐4 inhibitor groups exhibited reduced activity in the central area of the OFT, with a significant decrease in the total distance traveled (*p <* 0.01), a significant decrease in the percentage of sucrose preference (*p <* 0.01), and a significant prolongation of immobility time during the FST (*p <* 0.01) (Figure 4A–D).

Compared to the MOD group, the total distance traveled by mice in the EA and Combination groups in the OFT significantly increased (*p <* 0.01); sucrose preference significantly improved (*p <* 0.01 or *p <* 0.05), while immobility time during the FST significantly decreased (*p <* 0.01) (Figure 4A–D).

### 3.4 Electroacupuncture Decreases IL‐1β in the Serum and Increases IL‐4, IL‐10, and TGF‐β1 Levels in IBD Comorbid Depression Model Mice

**FIGURE 5:**
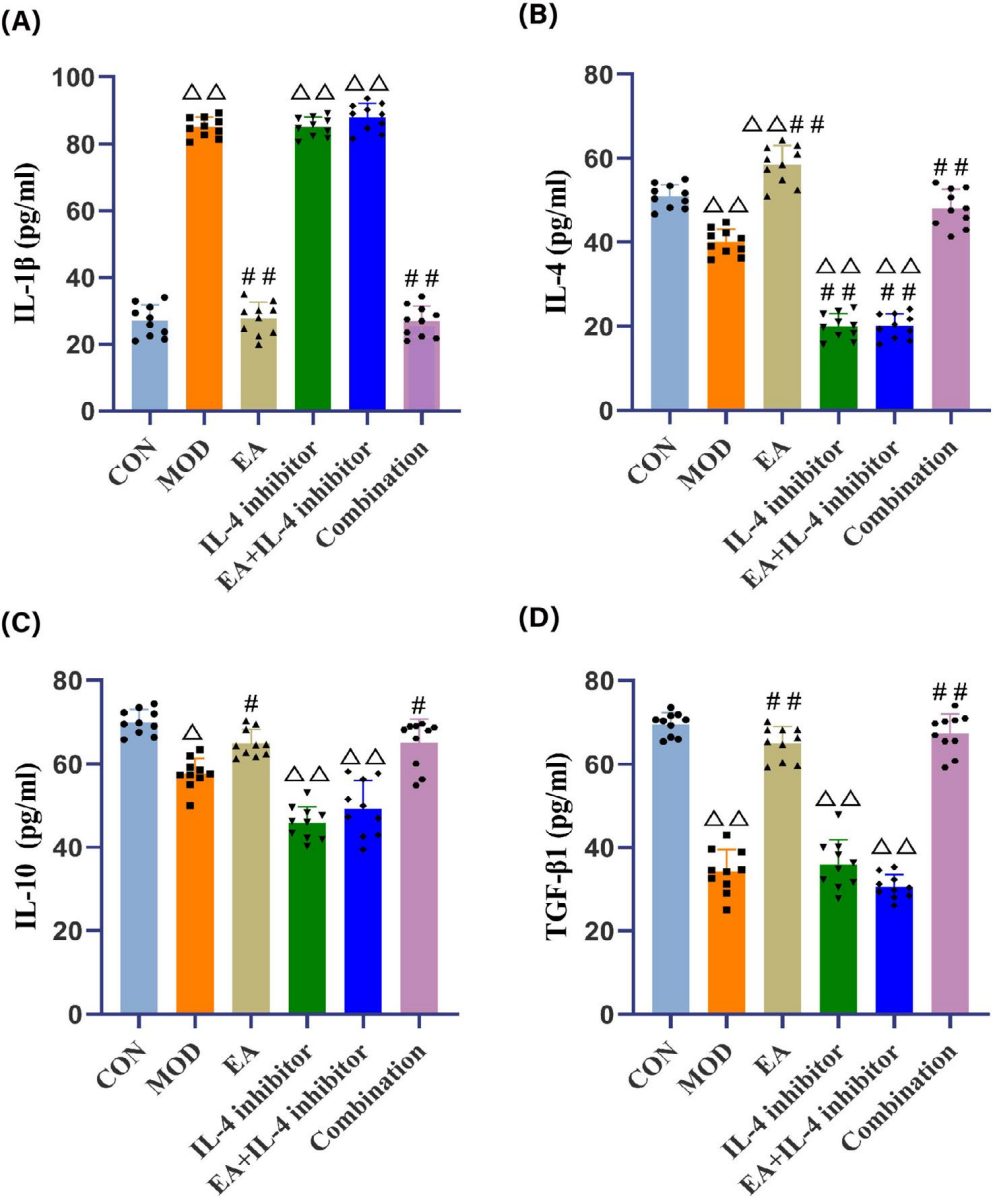
Serum concentration results of IL‐1β, IL‐4, IL‐10, and TGF‐β1 in mice of each group. (A) concentration results of serum IL‐1β. (B) Concentration results of serum IL‐4. (C) Concentration results of serum IL‐10. (D) Concentration results of serum TGF‐β1. *N* = 10 mice for each group. Compared with the CON group, ^△^ *p <* 0.05, ^△△^ *p <* 0.01; compared with the MOD group, ^#^ *p <* 0.05, ^##^ *p <* 0.01.

Compared to the CON group, serum IL‐1β levels in the MOD, IL‐4 inhibitor, and EA + IL‐4 inhibitor groups were significantly increased (*p <* 0.01); while IL‐4, IL‐10, and TGF‐β1 levels were significantly decreased (*p <* 0.01 or *p <* 0.05). Notably, serum IL‐4 levels in the EA group were significantly increased (*p <* 0.01) (Figure 5).

Compared to the MOD group, serum IL‐1β levels in the EA and Combination groups were significantly reduced (*p <* 0.01), while IL‐4, IL‐10, and TGF‐β1 levels were significantly increased (*p <* 0.01 or *p <* 0.05). Conversely, serum IL‐4 and IL‐10 levels in the IL‐4 inhibitor and the EA + IL‐4 inhibitor groups were significantly reduced (*p <* 0.01) (Figure 5).

### 3.5 Electroacupuncture Effectively Improves Neuroinflammation and Promotes Microglial M2 Polarization

**FIGURE 6:**
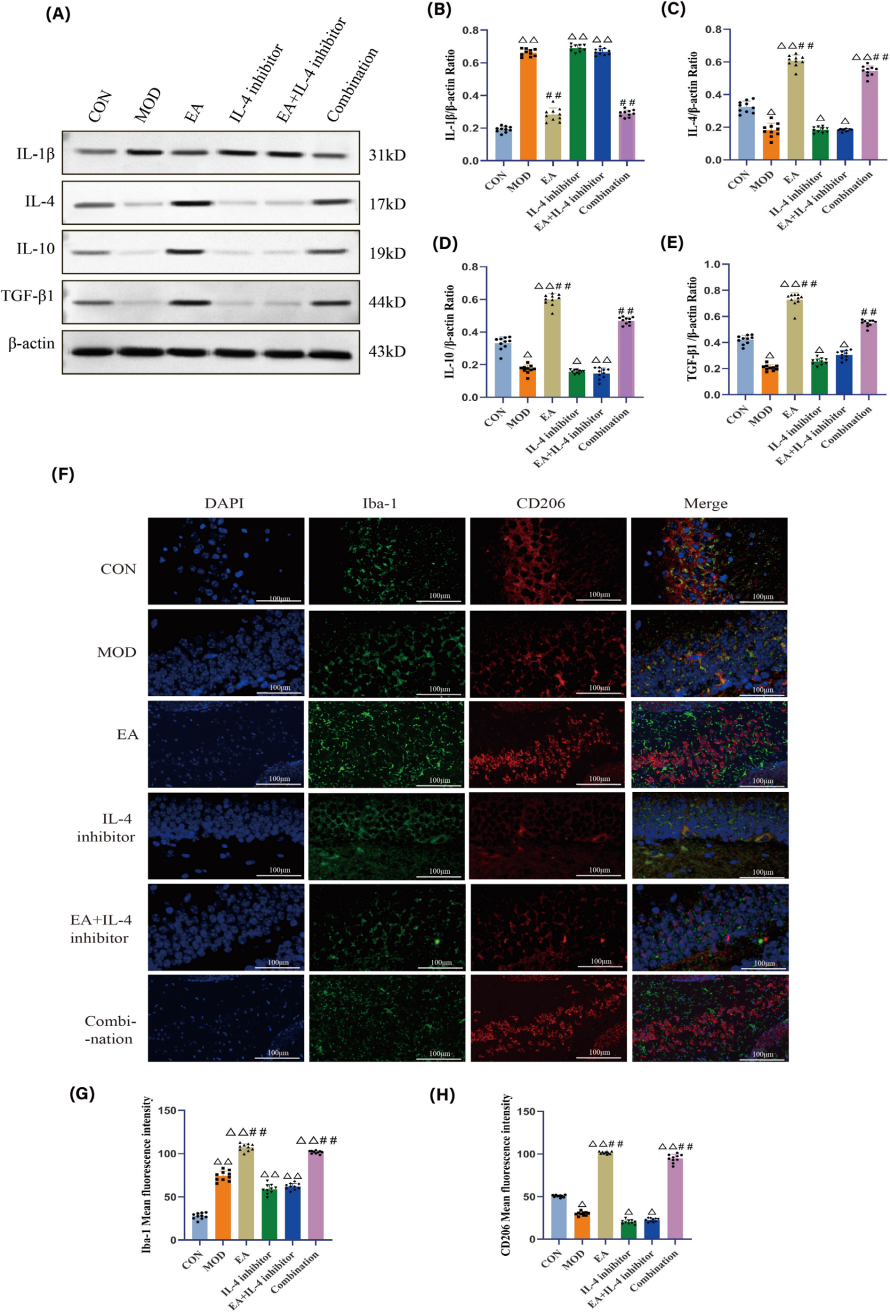
Detection results of central nervous system inflammation‐related indicators in mice of each group. (A–E) Protein expression levels of IL‐1β, IL‐4, IL‐10, and TGF‐β1 in the hippocampal tissue of mice in each group. (F) Expression of Iba‐1 and CD206 in the hippocampal region of mice in each group, with cell nuclei (blue), Iba‐1 (green), and CD206 (red). (G) Immunofluorescence intensity of Iba‐1 in the hippocampal region of mice in each group. (H) Immunofluorescence intensity of CD206 in the hippocampal region of mice in each group. *N* = 10 mice for each group. Compared with the CON group, ^△^ *p <* 0.05, ^△△^ *p <* 0.01; compared with the MOD group, ^##^ *p <* 0.01.

WB analysis revealed that, compared to the CON group, IL‐1β expression in the hippocampal tissue in the MOD, IL‐4 inhibitor, and EA + IL‐4 inhibitor groups significantly increased (*p <* 0.01); but was significantly lower in the EA and Combination groups than that in the MOD group (*p <* 0.01) (Figure 6A–E).

Compared to the CON group, IL‐4, IL‐10, and TGF‐β1 expression in the MOD, IL‐4 inhibitor, and EA + IL‐4 inhibitor groups significantly decreased (*p <* 0.05); but significantly increased in the EA and Combination groups (*p <* 0.01). Compared to the MOD group, IL‐4, IL‐10, and TGF‐β1 expression in the EA and Combination groups significantly increased (*p <* 0.01), indicating an improvement in the inflammatory environment (Figure 6A–E).

Compared to the CON group, the fluorescence intensity of Iba‐1 in hippocampal tissues in all other groups increased (*p <* 0.01); whereas the fluorescence intensity of CD206 in hippocampal tissues in the MOD, IL‐4 inhibitor, and EA + IL‐4 inhibitor groups significantly decreased (*p <* 0.05). Compared to the MOD group, the fluorescence intensity of Iba‐1 and CD206 in hippocampal tissues in the EA and Combination groups significantly increased (*p <* 0.01) (Figure 6F–H).

### 3.6 Electroacupuncture Regulates the IL‐4‐JAK1‐STAT6 Signaling Pathway to Achieve Therapeutic Effects in the Treatment of IBD Comorbid With Depression

**FIGURE 7:**
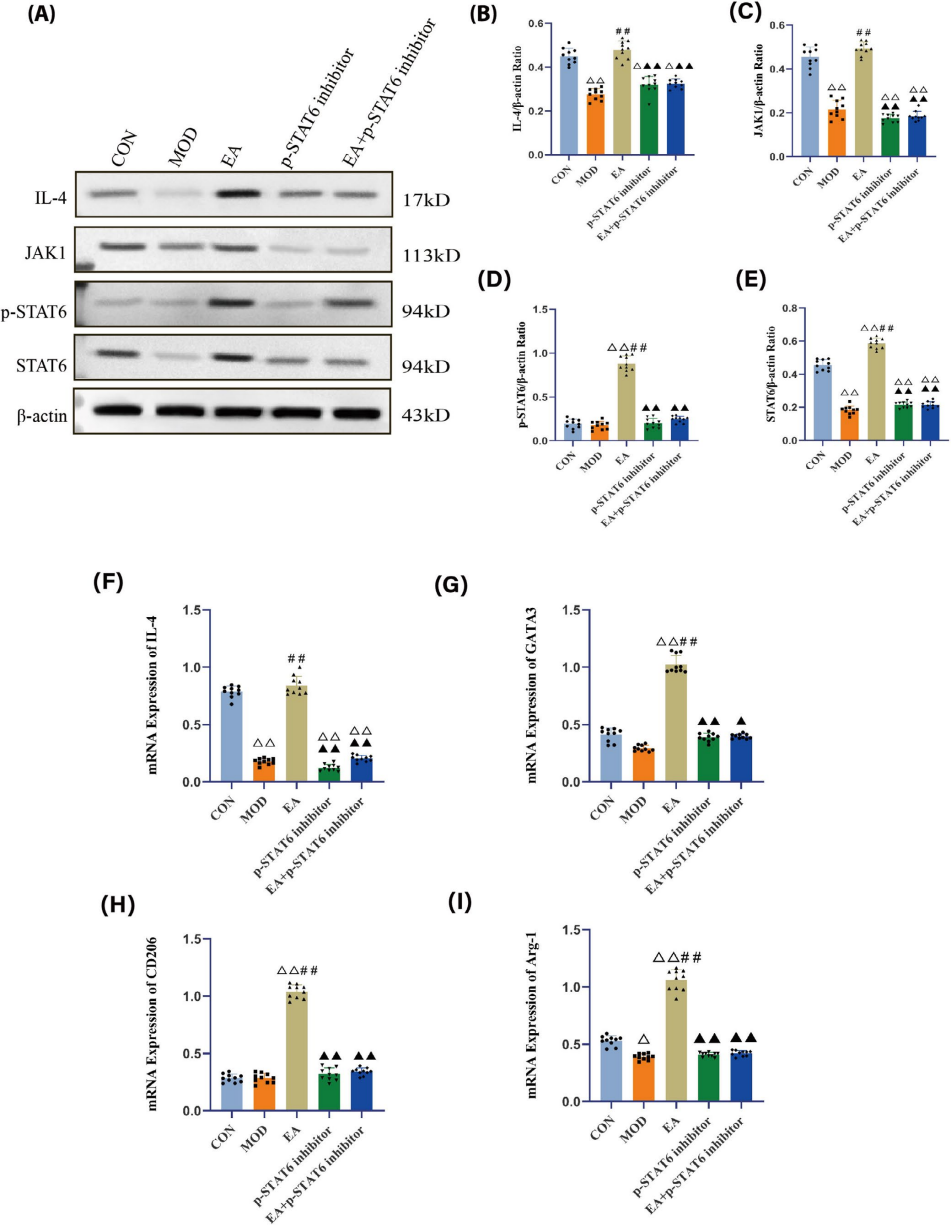
Detection results of IL‐4‐JAK1‐STAT6 pathway‐related indicators in mice of each group. (A–E) Protein expression levels of IL‐4, JAK1, p‐STAT6, and STAT6 in the hippocampal tissue of mice in each group. (F–I) Relative mRNA expression levels of IL‐4, GATA3, CD206, and Arg‐1 in the hippocampal tissue of mice in each group. *N* = 10 mice for each group. Compared with the CON group, ^△^ *p <* 0.05, ^△△^ *p <* 0.01; compared with the MOD group, ^##^ *p <* 0.01; compared with the EA group, ^▲^ *p <* 0.05, ^▲▲^ *p <* 0.01.

Compared to the CON group, p‐STAT6 and STAT6 expression in hippocampal tissues in the EA group significantly increased (*p <* 0.01), while IL‐4, JAK1, and STAT6 expression in the MOD, p‐STAT6 inhibitor, and EA + *p*‐STAT6 inhibitor groups significantly decreased (*p <* 0.01). Compared to the MOD group, IL‐4, JAK1, p‐STAT6, and STAT6 expression in hippocampal tissues in the EA group significantly increased (*p <* 0.01 or *p <* 0.05). Compared to the EA group, IL‐4, JAK1, p‐STAT6, and STAT6 expression in the p‐STAT6 inhibitor and EA + *p*‐STAT6 inhibitor groups significantly decreased (*p <* 0.01 or *p <* 0.05) (Figure 7A–E).

Compared to the CON group, GATA3 and CD206 mRNA expression in hippocampal tissues in the EA group significantly increased (*p <* 0.01); while the mRNA expression of IL‐4 in the MOD, p‐STAT6 inhibitor, and EA + *p*‐STAT6 inhibitor groups significantly decreased (*p <* 0.01). Compared to the MOD group, IL‐4, GATA3, CD206, and Arg‐1 mRNA expression in hippocampal tissues in the EA group significantly increased (*p <* 0.01 or *p <* 0.05). Compared to the EA group, IL‐4, GATA3, CD206, and Arg‐1 mRNA expression in the p‐STAT6 inhibitor and EA + *p*‐STAT6 inhibitor groups significantly decreased (*p <* 0.01 or *p <* 0.05) (Figure 7F–I).

**FIGURE 8:**
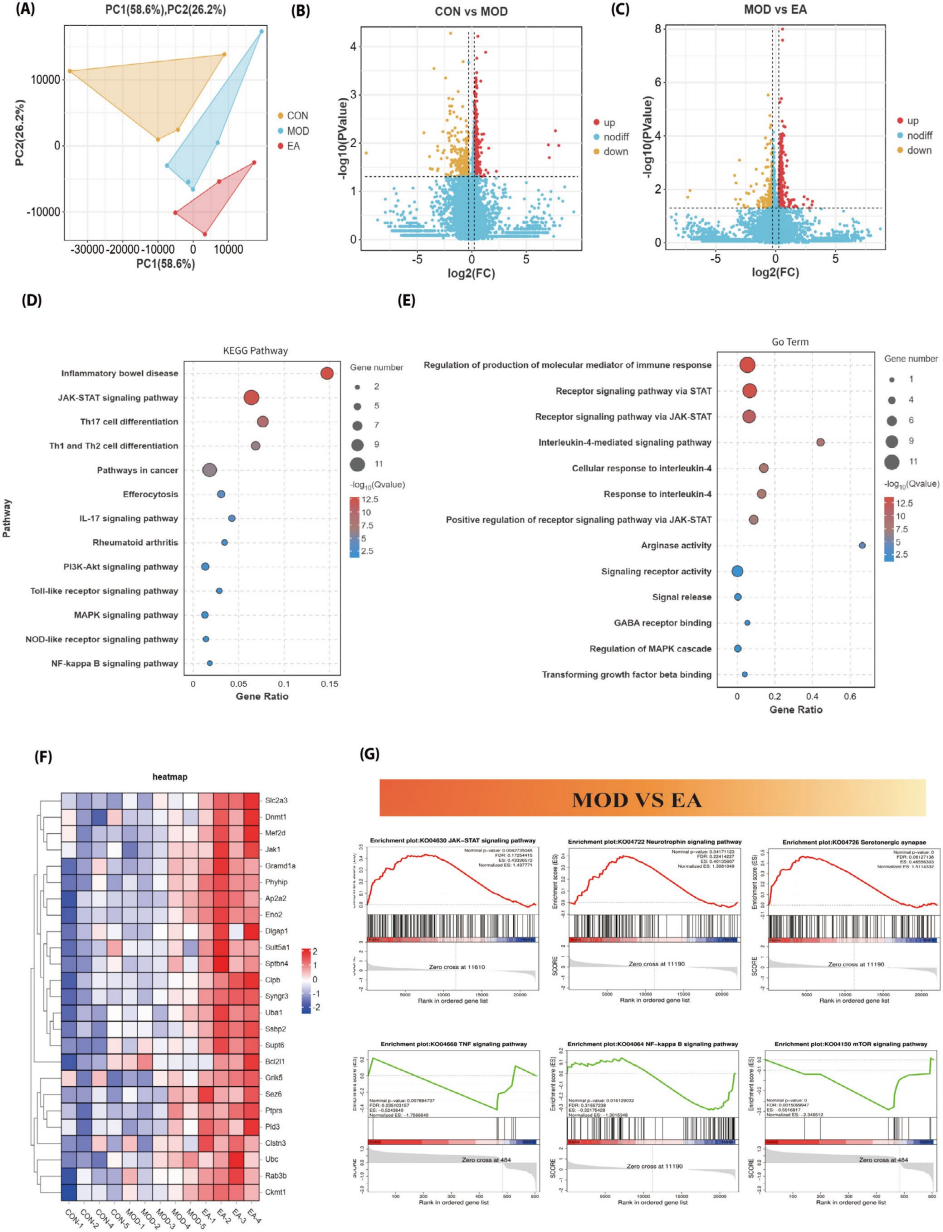
RNA‐seq results of hippocampal tissue in each group of mice. (A) PCA of the CON group, MOD group, and EA group. (B) Volcano plot of differentially expressed genes between the CON group and the MOD group. (C) Volcano plot of DEGs between the MOD group and the EA group. (D) KEGG enrichment analysis of DEGs between the MOD group and the EA group. (E) GO enrichment analysis of DEGs between the MOD group and the EA group. (F) Heatmap of DEGs in the CON group, MOD group, and EA group. (G) GSEA comparing the MOD group and the EA group.

The results of the hippocampal tissue transcriptome sequencing are shown in Figure 8. Principal component analysis (PCA) revealed significant differences in the transcriptome profiles among the three groups of mice (Figure 8A). Compared with the CON group, the MOD group upregulated 601 genes and downregulated 245 genes; compared with the MOD group, the EA group upregulated 598 genes and downregulated 241 genes (Figure 8B,C).

Compared with the MOD group, KEGG enrichment analysis indicated that the differentially expressed genes in the EA group were mainly associated with “inflammatory bowel disease,” “JAK‐STAT signaling pathway,” and “Th17 cell differentiation” (Figure 8D). GO enrichment analysis showed that the differentially expressed genes in the EA group were primarily related to “Regulation of production of molecular mediator of immune response,” “Receptor signaling pathway via STAT,” “Receptor signaling pathway via JAK‐STAT,” “Interleukin‐4‐mediated signaling pathway,” “Cellular response to IL‐4,” and “Positive regulation of receptor signaling pathways via JAK‐STAT” (Figure 8E).

The heatmap shows that neuroprotective‐related genes (such as Slc2a3, Mef2d, Jak1, and Syngr3) are highly expressed in the EA group (Figure 8F). GSEA results indicate that, compared with the MOD group, the JAK–STAT signaling pathway (NES = 1.44, *p* < 0.05), neurotrophin signaling pathway (NES = 1.31, *p* < 0.05), and serotonergic synapse (NES = 1.51, *p* < 0.05) are activated in the EA group; while the TNF signaling pathway (NES = −1.76, *p* < 0.05), NF‐κB signaling pathway (NES = −1.30, *p* < 0.05), and mTOR signaling pathway (NES = −2.35, *p* < 0.05) are inhibited (Figure 8G).

## 4 Discussion

**FIGURE 9:**
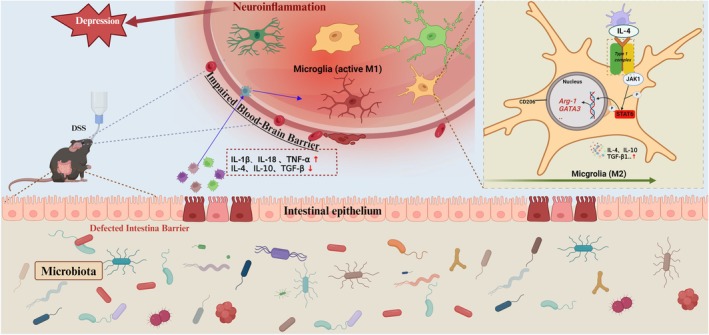
Inflammatory bowel disease and depression triggered by central nervous system inflammation, IL‐4‐JAK1‐STAT6 mediated M2 polarization of microglia.

This study revealed that electroacupuncture effectively modulates aberrant microglial activation, promotes the polarization of microglia towards the M2 phenotype, and alleviates neuroinflammation (Figure 9). Additionally, we identified the IL‐4‐JAK1‐STAT6 signaling as a crucial pathway implicated in facilitating these effects.

An increasing number of clinical studies have shown that electroacupuncture can effectively treat intestinal inflammatory responses and associated depressive symptoms in patients with IBD by regulating the release of inflammatory mediators in the body [33, 34, 35]. Animal research has further confirmed that electroacupuncture can improve neuroinflammation by inhibiting abnormal activation of microglia and regulating their polarization towards the M2 phenotype [36, 37]. Our findings align with these studies, indicating that electroacupuncture can confer therapeutic benefits in mice with IBD and concurrent depression.

Neuroinflammation serves as a significant pathological feature of IBD with comorbid depression, with aberrant microglial activation playing a pivotal role in this process (Figure 8) [38, 39, 40]. As the immune cells of the central nervous system, microglia achieve immune regulation through two polarization states, M1 and M2, under normal physiological conditions [12, 41]. In patients with IBD comorbid with depression, microglia are often in the M1 state, leading to the excessive release of pro‐inflammatory factors such as IL‐1β and tumor necrosis factor α, which further exacerbate central nervous system inflammation and trigger depressive symptoms [42, 43, 44]. Thus, inhibiting aberrant microglial activation and promoting their shift towards the M2 phenotype to ameliorate neuroinflammation represents a key therapeutic approach in the clinical management of IBD with depression.

Current treatment for IBD comorbid with depression relies mainly on medications that inhibit microglial activation and reduce inflammatory mediators to achieve antidepressant effects [45]. An example includes the combined use of 5‐aminosalicylic acid preparations such as mesalazine and antidepressant medications such as second‐generation tetracyclines and minocyclines. Kruis et al. [46] reported that the administration of the anti‐inflammatory drug mesalazine can suppress the secretion of pro‐inflammatory cytokines, including tumor necrosis factor‐α and interleukin‐6, from intestinal glands and immune cells, thereby effectively alleviating symptoms such as diarrhea and fecal blood in patients with IBD. Almradi et al. [47] revealed that ustekinumab selectively targets IL‐12 and IL‐23, preventing their interaction with their respective receptors, ultimately impeding the activation of downstream pro‐inflammatory pathways, thereby effectively alleviating inflammation in patients with IBD. Husain et al. [48] found that the anti‐inflammatory effect of minocycline may help improve depressive symptoms. These therapies exert their therapeutic effects by suppressing inflammatory responses. Although these treatments target inflammation, EA represents a non‐pharmacological alternative that leverages similar mechanisms to achieve therapeutic effects.

An increasing number of studies have shown that IL‐4 signaling plays a key role in microglial polarization and mediates neuroinflammatory responses, operating a regulatory effect on microglia via the JAK1‐STAT6 pathway. Upon binding to its receptor, IL‐4 triggers the formation of a type I signaling complex, which facilitates the phosphorylation of tyrosine residues on JAK1. This process subsequently generates a docking site for STAT6. Subsequently, STAT6 undergoes phosphorylation and dimerization and is translocated to the nucleus, facilitating the transcription of GATA3 (a Th2 cell‐inducing factor), thereby driving microglia towards an anti‐inflammatory M2 phenotype [49, 50]. During this process, the M2 marker Arg‐1 in microglia significantly increases [51], and anti‐inflammatory factors such as IL‐4, IL‐10, and TGF‐β1 are released (Figure 8). Therefore, IL‐4 is mainly responsible for the polarization of microglia to the M2 phenotype via the JAK1‐STAT6 pathway [14].

CD206 is a specific marker for M2‐type microglia, and its function plays an important role in regulating immune responses [52]. Increased expression of GATA3 is associated with the expression of anti‐inflammatory genes of M2‐type microglia, such as Arg‐1. The elevated expression of these genes plays a crucial role in mitigating neuroinflammation [12]. Arg‐1, in particular, serves as a key indicator of M2‐type microglia and can suppress neuroinflammation by facilitating the shift of microglia from the pro‐inflammatory M1 phenotype to the anti‐inflammatory M2 phenotype [53].

Our investigation revealed that DSS‐induced IBD mice experienced weight loss, significantly elevated DAI scores, and overt depressive‐like behaviors, including lethargic movement and diminished activity levels. These findings align with those from prior studies [54], suggesting that IBD is not merely a gastrointestinal disorder but is also intimately connected to mental and emotional health. Following electroacupuncture therapy, the mice exhibited marked improvement in general condition and depressive‐like behaviors, a substantial reduction in inflammatory cell infiltration within the intestines, and partial restoration of mucosal damage. The concentrations of IL‐4, IL‐10, and TGF‐β1 in both the serum and colon tissues were markedly elevated, whereas the concentration of IL‐1β was notably reduced. Additionally, the expression of JAK1, p‐STAT6, and STAT6 within hippocampal tissue was increased. A considerable change in the expression of the M2‐type marker CD206 was observed in the mice, along with a significant increase in the expression of IL‐4, Arg‐1, CD206, and GATA3 mRNA. This indicates that the IL‐4‐JAK1‐STAT6 signaling pathway may be a crucial mechanism through which electroacupuncture suppresses the aberrant activation of microglia, encourages their shift towards the M2 phenotype, and alleviates neuroinflammation in the treatment of IBD comorbid with depression. Upon further validation using groups treated with an IL‐4 inhibitor and a p‐STAT6 inhibitor, we observed that the level of IL‐4 in IBD model mice exhibiting depression significantly decreased, whereas neuroinflammation did not show improvement. This further demonstrates that the IL‐4‐JAK1‐STAT6 signaling pathway could indeed be a crucial mechanism through which electroacupuncture modulates the aberrant activation of microglia and ameliorates neuroinflammation. Subsequently, we performed RNA‐seq on the hippocampal tissue of mice, and the analysis indicated that electroacupuncture improves IBD with depression by inhibiting neuroinflammation through activation of the IL‐4‐JAK1‐STAT6 signaling pathway.

The symptoms of IBD with depression typically manifest as liver stagnation and spleen deficiency, with inadequate liver qi release and spleen qi insufficiency collectively underlying the pathogenesis. These two conditions frequently exacerbate each other, resulting in impaired function of the large intestine, obstruction of qi and blood circulation, and damage to the intestinal collaterals. As such, treatment should use the liver and spleen as the primary access points [55]. In this study, Zusanli, Tianshu, and Taichong were selected as the therapeutic acupoints for intervention. Zusanli and Tianshu are frequently utilized in acupuncture treatment for IBD, while Taichong is particularly significant for treating depressive disorders [56, 57]. The synergistic application of these three acupoints enhances the body's resistance, eliminates pathogenic elements, soothes the liver, alleviates depression, and fortifies the spleen to prevent diarrhea.

## 5 Conclusion

In summary, this study found that electroacupuncture regulates the IL‐4‐JAK1‐STAT6 signaling pathway, inhibits abnormal microglia activation, promotes their polarization towards the M2 phenotype, alleviates neuroinflammation, and improves depressive‐like behaviors in mice with IBD and comorbid depression. This finding provides a scientific basis for using electroacupuncture to treat depressive symptoms in patients with IBD. However, during the research process, we found that the effects of electroacupuncture depend on the synergistic actions of multiple pathways and mechanisms. Therefore, in our future studies, we will explore other pathways through which electroacupuncture inhibits neuroinflammation to treat IBD with depression, based on the RNA‐seq results. We aim to clarify the interrelationships between these pathways and provide a scientific basis for the treatment of IBD with depression using electroacupuncture.

## Author Contributions

S.C.: conceptualization, project administration, writing‐original draft, writing‐review and editing. J.Y.: conceptualization, project administration, writing‐original draft. L.C.: project administration, data section, writing‐original draft. Z.L.: conceptualization, project administration, and writing. L.J.: conceptualization, project administration, and writing. Y.H.: project administration. Z.X.: project administration. P.L.: project administration. J.L.: conceptualization, project administration. Q.L.: conceptualization, project administration, formal analysis. M.L.: conceptualization, review and editing, and funding acquisition.

## Conflicts of Interest

The authors declare no conflicts of interest.

## Supporting information


**Data S1:** cns70572‐sup‐0001‐Supinfo.pdf.

## Data Availability

The data that support the findings of this study are available from the corresponding author upon reasonable request.
