# Supplementary material for: IL‐4‐JAK1‐STAT6 Pathway Mediates Electroacupuncture's Effect on Microglial M2 Polarization to Treat Inflammatory Bowel Disease With Comorbid Depression
Source: CNS Neurosci Ther. 2025 Aug 18;31(8):e70572. doi: 10.1111/cns.70572 (PMC12358807; doi:10.1111/cns.70572)
Supplement: Supplementary file 1 — Data S1: cns70572‐sup‐0001‐Supinfo.pdf. [file CNS-31-e70572-s001.pdf]

Supplementary data Original blots

For fig2（C）. Expression levels of IL-1β in the colon tissue of mice from the CON group and DSS group post-model induction.

1. IL-1β (30kDa). For Figure 2 (C), a replicate of the experimental data shown in the manuscript is provided. Full unedited gel/blot for Figure 2 (C)

From left to right in order: Control/MOD/ EA/IL-4 inhibitor/EA+IL-4 inhibitor/Combination

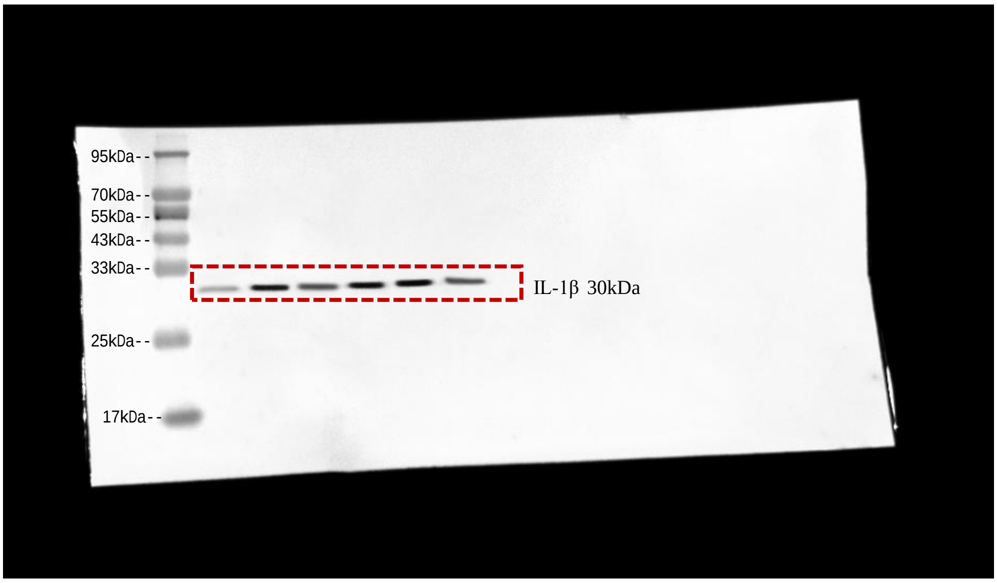

Control: Control group
MOD: Dextran sodium sulfate model group
EA: Electroacupuncture group
IL-4 inhibitor: Interleukin-4 Inhibitor Injection Group
EA + IL-4 inhibitor: Concurrent Electroacupuncture Treatment with Interleukin-4 Inhibitor Injection
Combination: Intraperitoneally injected with 5-aminosalicylic acid and minocycline

2. β-actin (43kDa). For Figure 2 (C), a replicate of the experimental data shown in the manuscript is provided. Full unedited gel/blot for Figure 2 (C)

From left to right in order: Control/MOD/ EA/IL-4 inhibitor/EA+IL-4 inhibitor/Combination

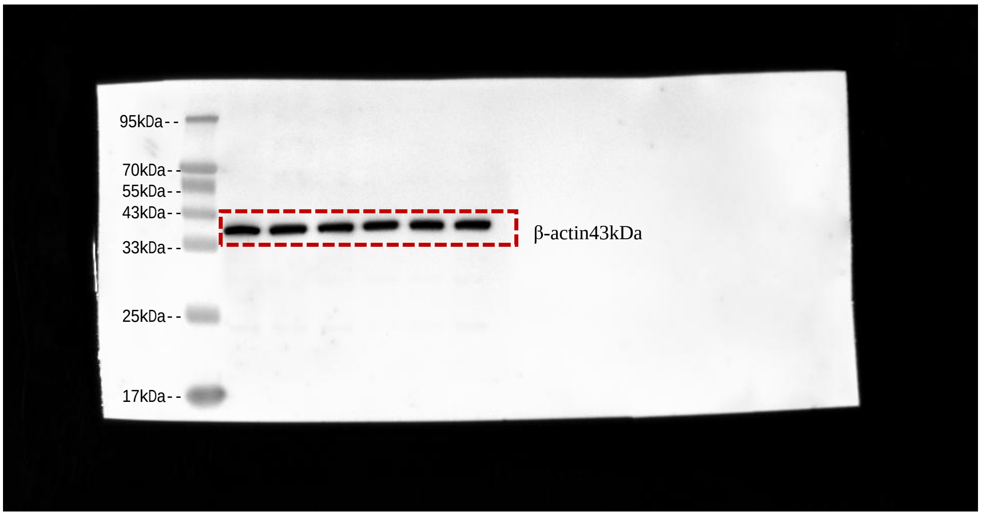

Control: Control group
MOD: Dextran sodium sulfate model group
EA: Electroacupuncture group
IL-4 inhibitor: Interleukin-4 Inhibitor Injection Group
EA + IL-4 inhibitor: Concurrent Electroacupuncture Treatment with Interleukin-4 Inhibitor Injection
Combination: Intraperitoneally injected with 5-aminosalicylic acid and minocycline

For fig3F. Protein expression levels of IL-1β, IL-4, IL-10, and TGF-β1 in colon tissue of each group of mice.

1. IL-1β(30kDa). For Figure 3F, a replicate of the experimental data shown in the manuscript is provided. Full unedited gel/blot for Figure 3 (F)

From left to right in order: Control/MOD/ EA/IL-4 inhibitor/EA+IL-4 inhibitor/Combination

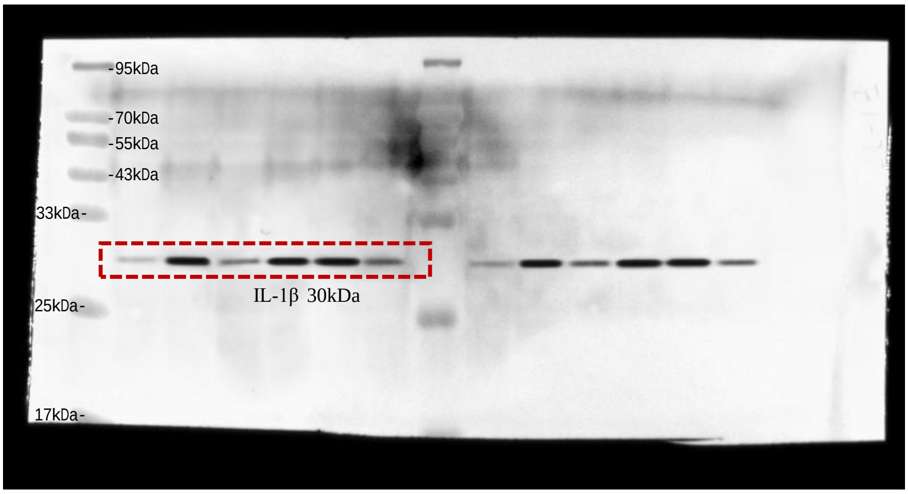

Control: Control group
MOD: Dextran sodium sulfate model group
EA: Electroacupuncture group
IL-4 inhibitor: Interleukin-4 Inhibitor Injection Group
EA + IL-4 inhibitor: Concurrent Electroacupuncture Treatment with Interleukin-4 Inhibitor Injection
Combination: Intraperitoneally injected with 5-aminosalicylic acid and minocycline

2. IL-4(30kDa). For Figure 3F, a replicate of the experimental data shown in the manuscript is provided. Full unedited gel/blot for Figure 3 (F)

From left to right in order: Control/MOD/ EA/IL-4 inhibitor/EA+IL-4 inhibitor/Combination

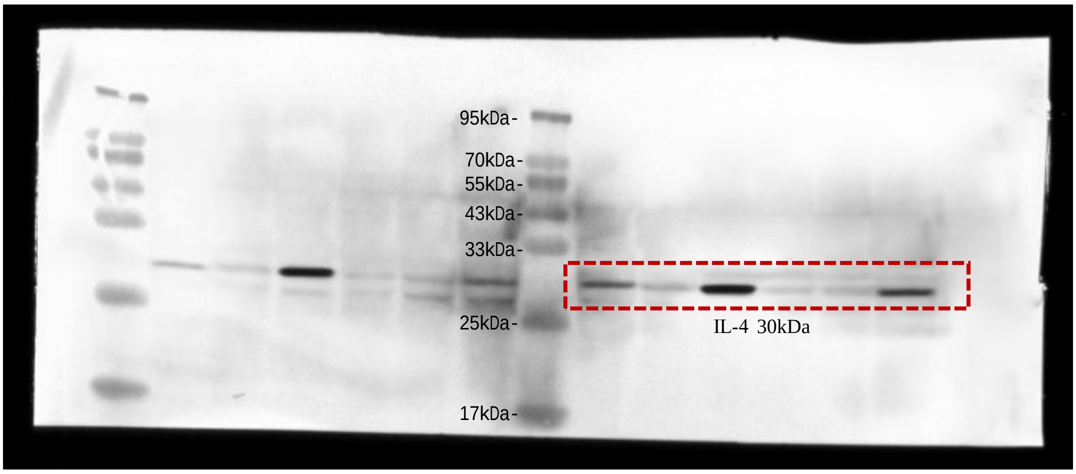

Control: Control group
MOD: Dextran sodium sulfate model group
EA: Electroacupuncture group
IL-4 inhibitor: Interleukin-4 Inhibitor Injection Group
EA + IL-4 inhibitor: Concurrent Electroacupuncture Treatment with Interleukin-4 Inhibitor Injection
Combination: Intraperitoneally injected with 5-aminosalicylic acid and minocycline

3. IL-10(21kDa). For Figure 3F, a replicate of the experimental data shown in the manuscript is provided. Full unedited gel/blot for Figure 3 (F)

From left to right in order: Control/MOD/ EA/IL-4 inhibitor/EA+IL-4 inhibitor/Combination

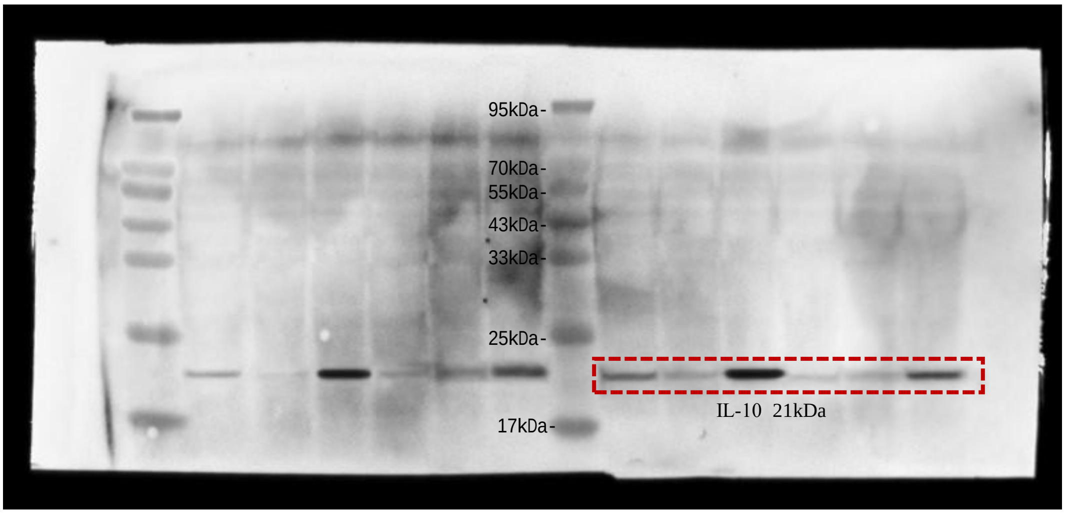

Control: Control group
MOD: Dextran sodium sulfate model group
EA: Electroacupuncture group
IL-4 inhibitor: Interleukin-4 Inhibitor Injection Group
EA + IL-4 inhibitor: Concurrent Electroacupuncture Treatment with Interleukin-4 Inhibitor Injection
Combination: Intraperitoneally injected with 5-aminosalicylic acid and minocycline

4. TGF-β1(44kDa). For Figure 3F, a replicate of the experimental data shown in the manuscript is provided. Full unedited gel/blot for Figure 3 (F)

From left to right in order: Control/MOD/ EA/IL-4 inhibitor/EA+IL-4 inhibitor/Combination

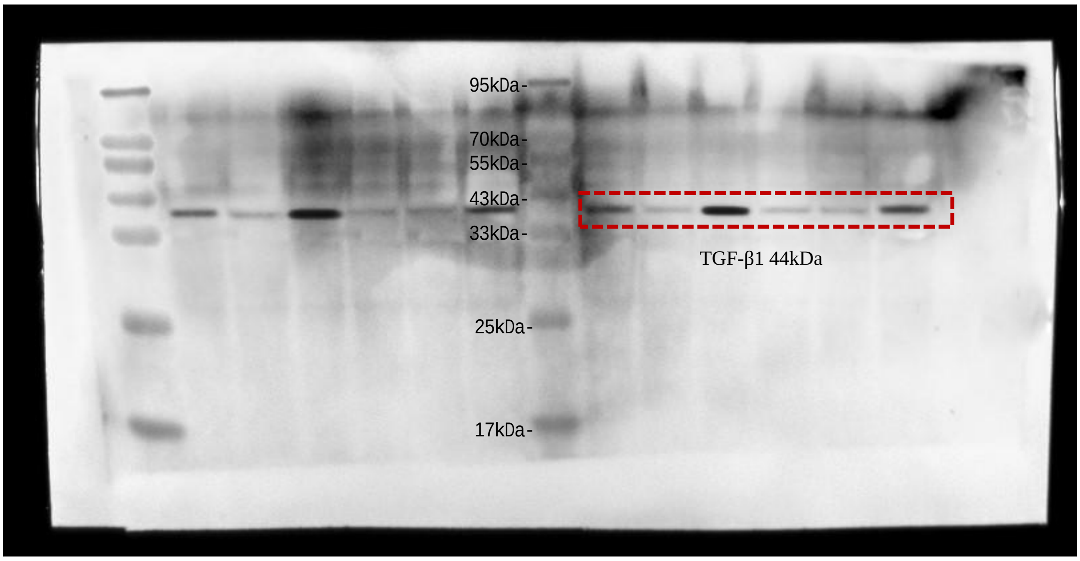

Control: Control group

MOD: Dextran sodium sulfate model group

EA: Electroacupuncture group

IL-4 inhibitor: Interleukin-4 Inhibitor Injection Group

EA + IL-4 inhibitor: Concurrent Electroacupuncture Treatment with Interleukin-4 Inhibitor Injection

Combination: Intraperitoneally injected with 5-aminosalicylic acid and minocycline

5. β-actin(43kDa). For Figure 3F, a replicate of the experimental data shown in the manuscript is provided. Full unedited gel/blot for Figure 3 (F)

From left to right in order: Control/MOD/ EA/IL-4 inhibitor/EA+IL-4 inhibitor/Combination

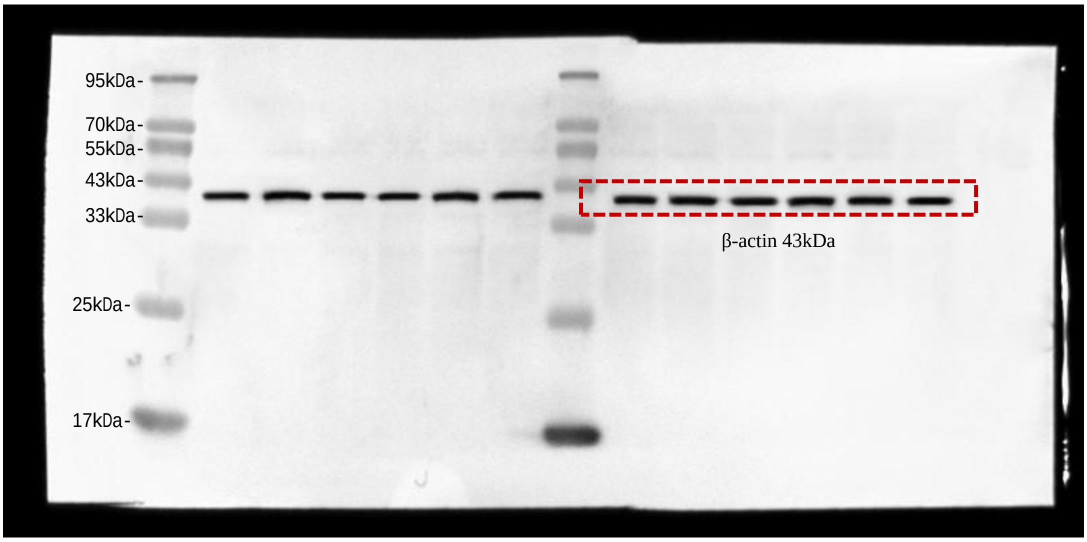

Control: Control group

MOD: Dextran sodium sulfate model group

EA: Electroacupuncture group

IL-4 inhibitor: Interleukin-4 Inhibitor Injection Group

EA + IL-4 inhibitor: Concurrent Electroacupuncture Treatment with Interleukin-4 Inhibitor Injection

Combination: Intraperitoneally injected with 5-aminosalicylic acid and minocycline

For fig6A. Protein expression levels of IL-1 β , IL-4, IL-10, and TGF- β 1 in the hippocampal tissue of mice in each group.

1.IL-1β(31kDa). For Figure 6A, a replicate of the experimental data shown in the manuscript is provided. Full unedited gel/blot for Figure 6 (A)

From left to right in order: Control/MOD/ EA/IL-4 inhibitor/EA+IL-4 inhibitor/Combination

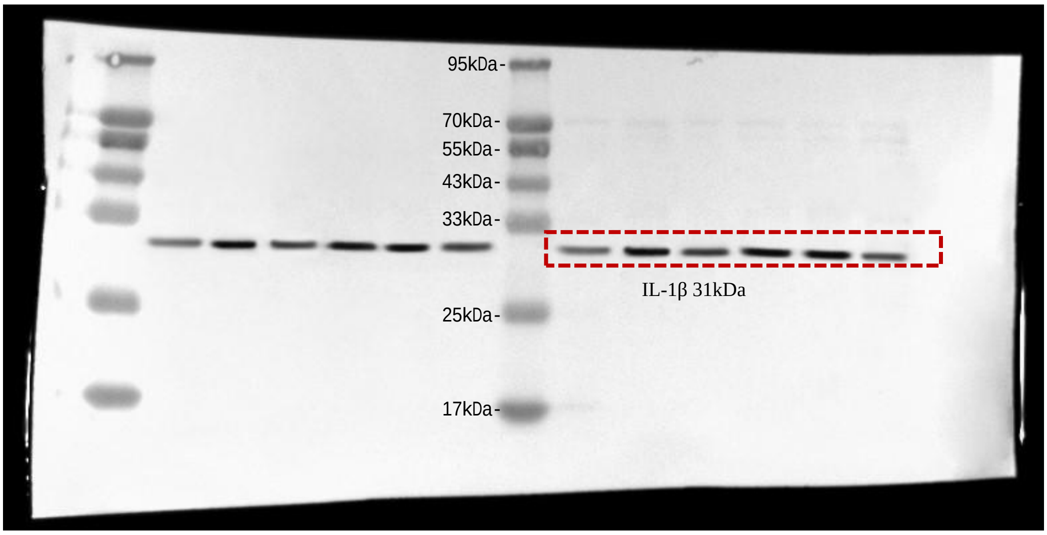

Control: Control group

MOD: Dextran sodium sulfate model group

EA: Electroacupuncture group

IL-4 inhibitor: Interleukin-4 Inhibitor Injection Group

EA + IL-4 inhibitor: Concurrent Electroacupuncture Treatment with Interleukin-4 Inhibitor Injection

Combination: Intraperitoneally injected with 5-aminosalicylic acid and minocycline

3. IL-4(17kDa). For Figure6(A), a replicate of the experimental data shown in the manuscript is provided. Full unedited gel/blot for Figure 6 (A)

From left to right in order: Control/MOD/ EA/IL-4 inhibitor/EA+IL-4 inhibitor/Combination

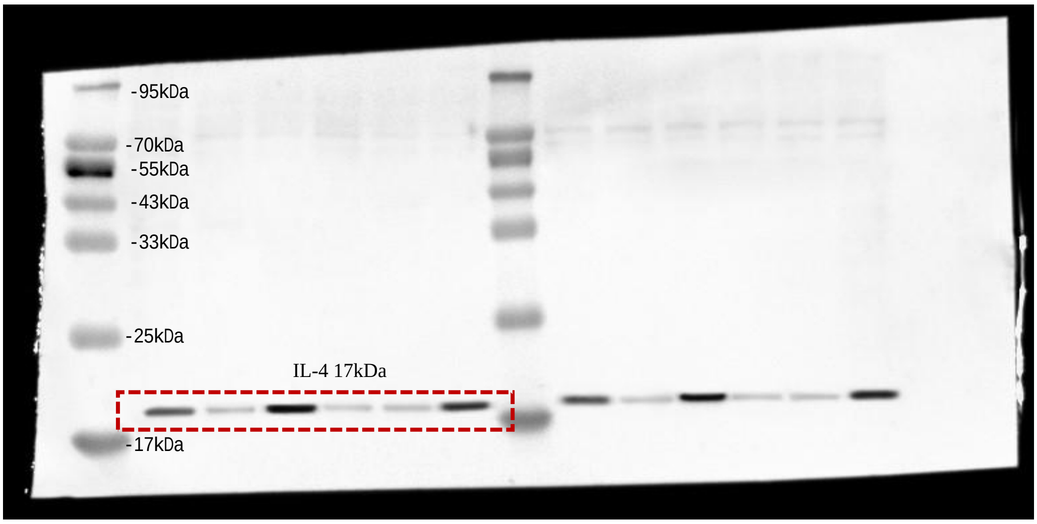

Control: Control group

MOD: Dextran sodium sulfate model group

EA: Electroacupuncture group

IL-4 inhibitor: Interleukin-4 Inhibitor Injection Group

EA + IL-4 inhibitor: Concurrent Electroacupuncture Treatment with Interleukin-4 Inhibitor Injection

Combination: Intraperitoneally injected with 5-aminosalicylic acid and minocycline

3.IL-10(19kDa). For Figure 6(A), a replicate of the experimental data shown in the manuscript is provided. Full unedited gel/blot for Figure 6 (A)

From left to right in order: Control/MOD/ EA/IL-4 inhibitor/EA+IL-4 inhibitor/Combination

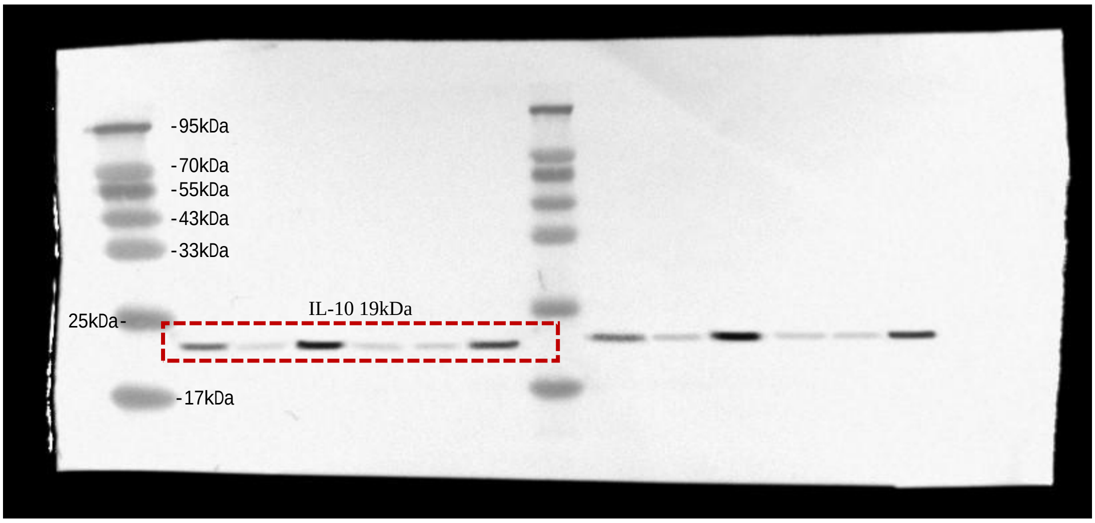

Control: Control group

MOD: Dextran sodium sulfate model group

EA: Electroacupuncture group

IL-4 inhibitor: Interleukin-4 Inhibitor Injection Group

EA + IL-4 inhibitor: Concurrent Electroacupuncture Treatment with Interleukin-4 Inhibitor Injection

Combination: Intraperitoneally injected with 5-aminosalicylic acid and minocycline

4.TGF-β1(44kDa). For Figure 6(A), a replicate of the experimental data shown in the manuscript is provided. Full unedited gel/blot for Figure 6 (A)

From left to right in order: Control/MOD/ EA/IL-4 inhibitor/EA+IL-4 inhibitor/Combination

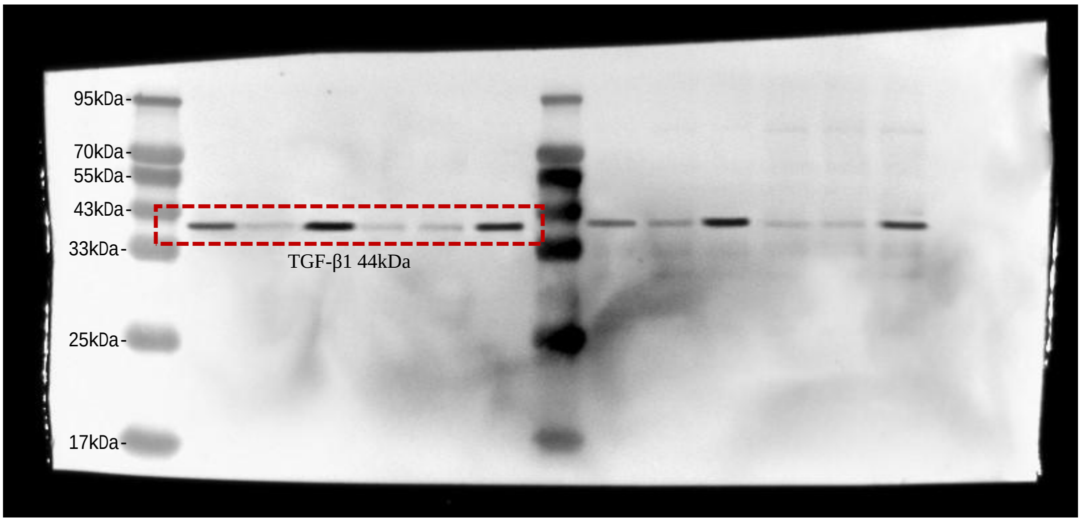

Control: Control group

MOD: Dextran sodium sulfate model group

EA: Electroacupuncture group

IL-4 inhibitor: Interleukin-4 Inhibitor Injection Group

EA + IL-4 inhibitor: Concurrent Electroacupuncture Treatment with Interleukin-4 Inhibitor Injection

Combination: Intraperitoneally injected with 5-aminosalicylic acid and minocycline

5. β-actin(43kDa). For Figure 6(A), a replicate of the experimental data shown in the manuscript is provided. Full unedited gel/blot for Figure 6 (A)

From left to right in order: Control/MOD/ EA/IL-4 inhibitor/EA+IL-4 inhibitor/Combination

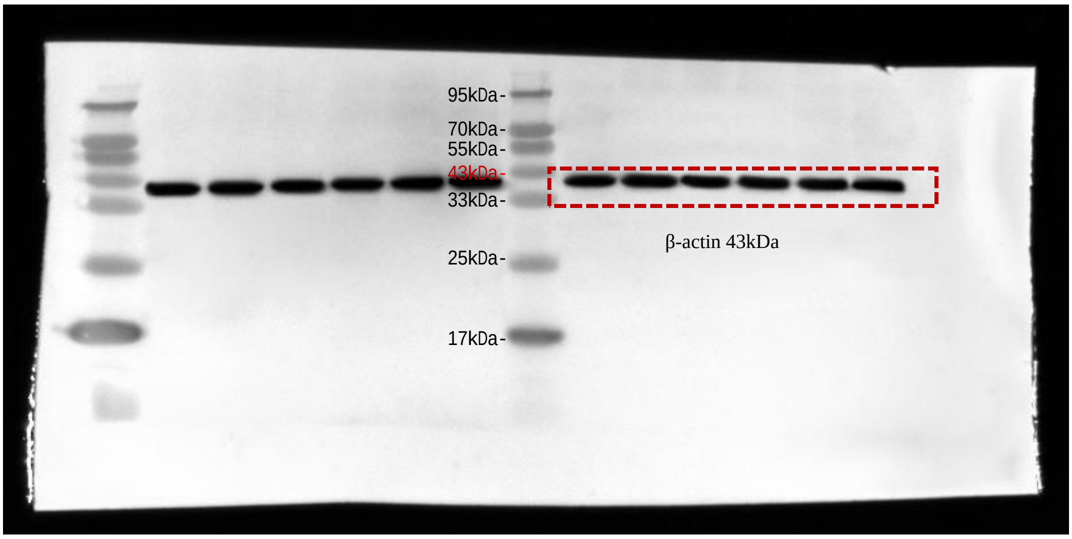

Control: Control group
MOD: Dextran sodium sulfate model group
EA: Electroacupuncture group
IL-4 inhibitor: Interleukin-4 Inhibitor Injection Group
EA + IL-4 inhibitor: Concurrent Electroacupuncture Treatment with Interleukin-4 Inhibitor Injection
Combination: Intraperitoneally injected with 5-aminosalicylic acid and minocycline

For fig7A. Protein expression levels of IL-4, JAK1, p-STAT6, and STAT6 in the hippocampal tissue of mice in each group. .

1. IL-4(17kDa). For Figure 7A, a replicate of the experimental data shown in the manuscript is provided. Full unedited gel/blot for Figure 7 (A)

From left to right in order: Control/MOD/ EA/p-STAT6 inhibitor/EA+p-STAT6 inhibitor

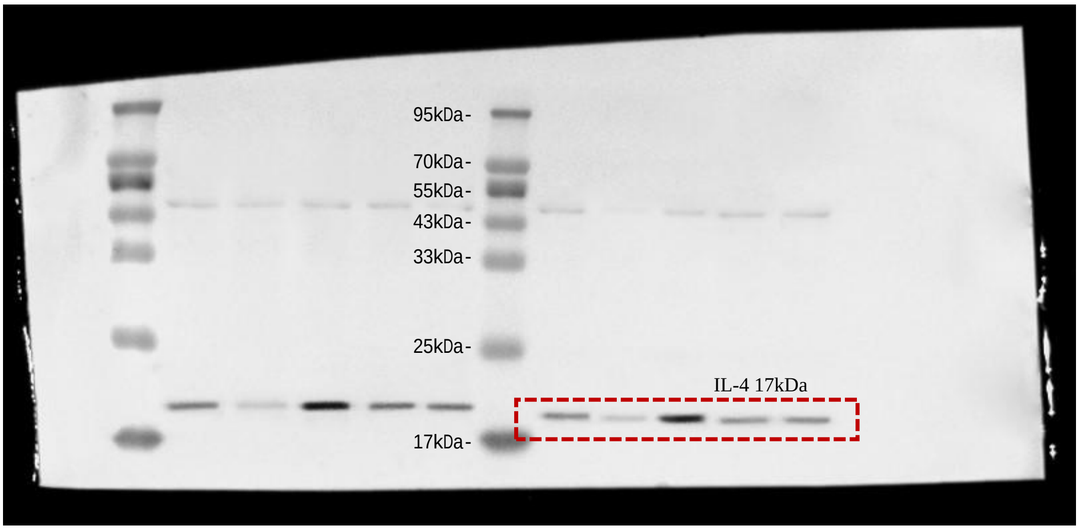

Control: Control group

MOD: Dextran sodium sulfate model group

EA: Electroacupuncture group

IL-4 inhibitor: p-STAT6 Inhibitor Injection Group

EA + IL-4 inhibitor: Concurrent Electroacupuncture Treatment with p-STAT6 Inhibitor Injection

2. JAK1(113kDa). For Figure 7A, a replicate of the experimental data shown in the manuscript is provided. Full unedited gel/blot for Figure 7 (A)

From left to right in order: Control/MOD/ EA/p-STAT6 inhibitor/EA+p-STAT6 inhibitor

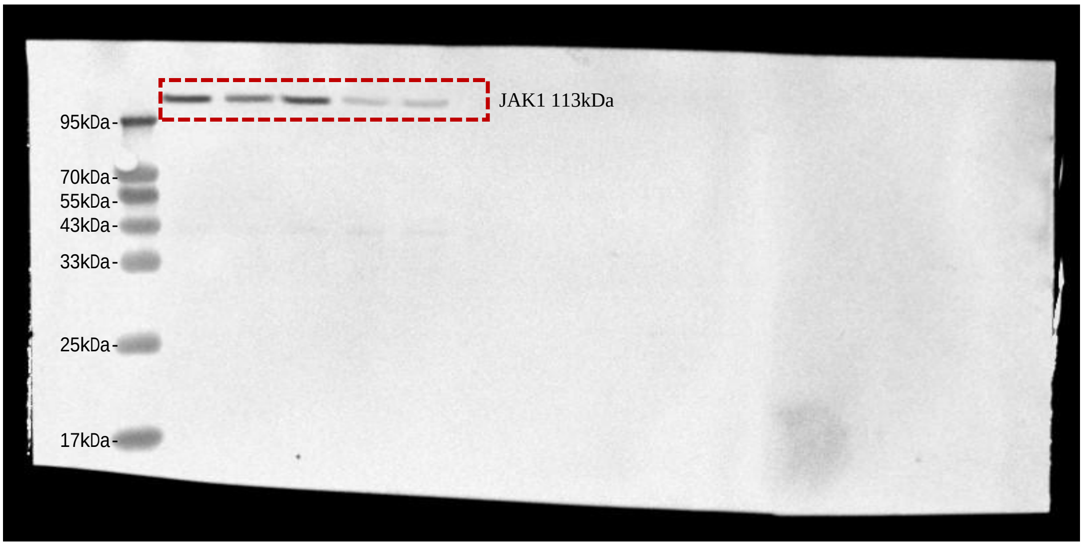

Control: Control group

MOD: Dextran sodium sulfate model group

EA: Electroacupuncture group

IL-4 inhibitor: p-STAT6 Inhibitor Injection Group

EA + IL-4 inhibitor: Concurrent Electroacupuncture Treatment with p-STAT6 Inhibitor Injection

3. p-STAT6(94kDa). For Figure 7A, a replicate of the experimental data shown in the manuscript is provided. Full unedited gel/blot for Figure 7 (A)

From left to right in order: Control/MOD/ EA/p-STAT6 inhibitor/EA+p-STAT6 inhibitor

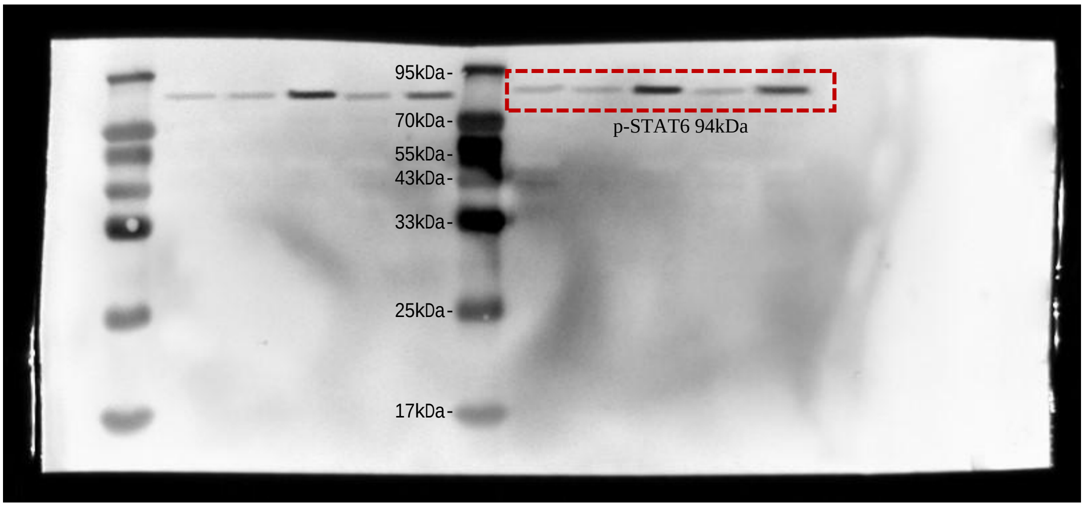

Control: Control group

MOD: Dextran sodium sulfate model group

EA: Electroacupuncture group

IL-4 inhibitor: p-STAT6 Inhibitor Injection Group

EA + IL-4 inhibitor: Concurrent Electroacupuncture Treatment with p-STAT6 Inhibitor Injection

4. STAT6(94kDa). For Figure 7A, a replicate of the experimental data shown in the manuscript is provided. Full unedited gel/blot for Figure 7 (A)

From left to right in order: Control/MOD/ EA/p-STAT6 inhibitor/EA+p-STAT6 inhibitor

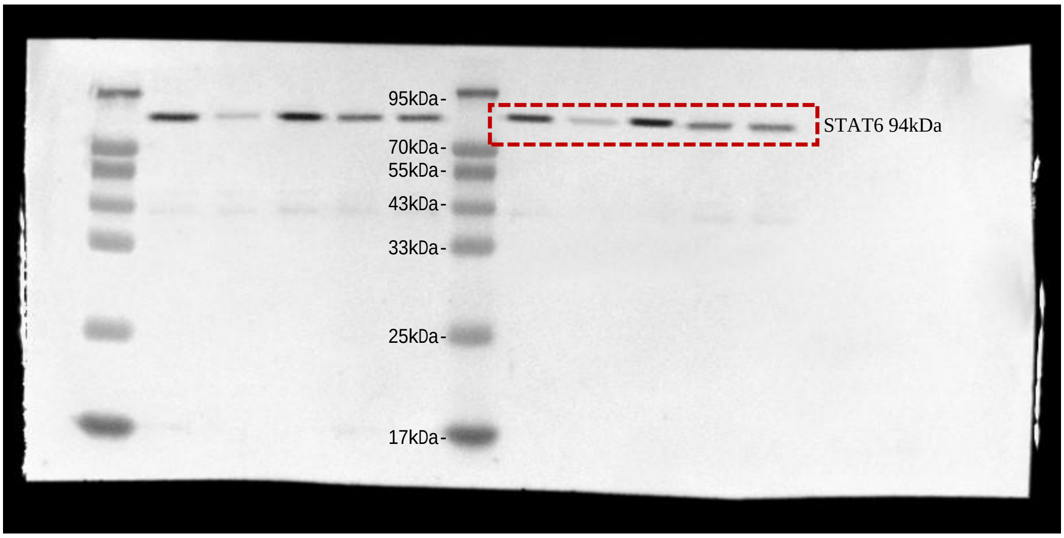

Control: Control group

MOD: Dextran sodium sulfate model group

EA: Electroacupuncture group

IL-4 inhibitor: p-STAT6 Inhibitor Injection Group

EA + IL-4 inhibitor: Concurrent Electroacupuncture Treatment with p-STAT6 Inhibitor Injection

5. β-actin(43kDa). For Figure 7A, a replicate of the experimental data shown in the manuscript is provided. Full unedited gel/blot for Figure 7 (A)

From left to right in order: Control/MOD/ EA/p-STAT6 inhibitor/EA+p-STAT6 inhibitor

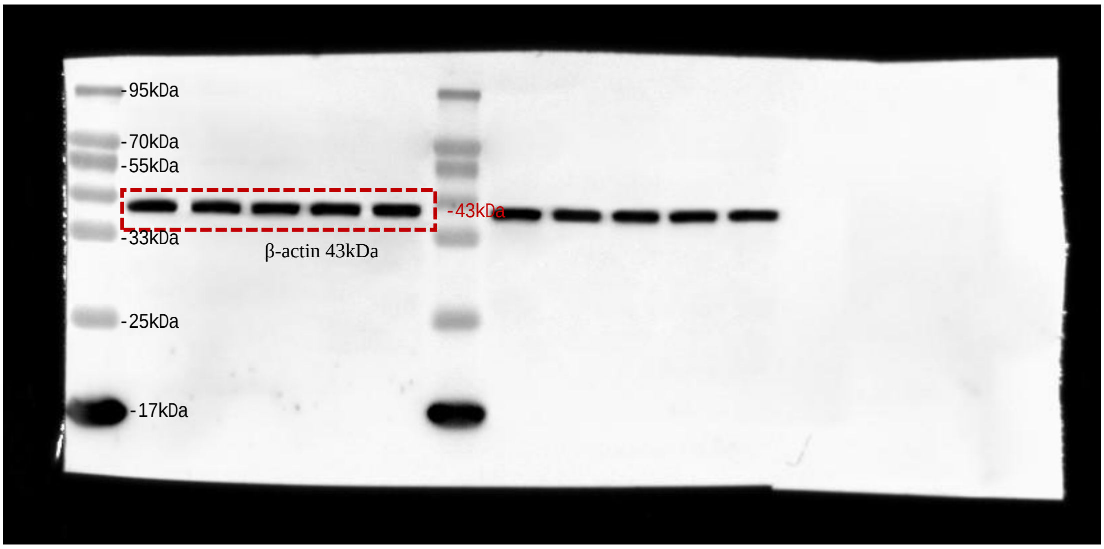

Control: Control group

MOD: Dextran sodium sulfate model group

EA: Electroacupuncture group

IL-4 inhibitor: p-STAT6 Inhibitor Injection Group

EA + IL-4 inhibitor: Concurrent Electroacupuncture Treatment with p-STAT6 Inhibitor Injection
